# A Predictive Maintenance Model for Flexible Manufacturing in the Context of Industry 4.0

**DOI:** 10.3389/fdata.2021.663466

**Published:** 2021-08-25

**Authors:** Go Muan Sang, Lai Xu, Paul de Vrieze

**Affiliations:** Faculty of Science and Technology, Bournemouth University, Poole, United Kingdom

**Keywords:** Industry 4.0, predictive maintenance, big data analytics, maintenance schedule plan, flexible manufacturing

## Abstract

The Industry 4.0 paradigm is the focus of modern manufacturing system design. The integration of cutting-edge technologies such as the Internet of things, cyber–physical systems, big data analytics, and cloud computing requires a flexible platform supporting the effective optimization of manufacturing-related processes, e.g., predictive maintenance. Existing predictive maintenance studies generally focus on either a predictive model without considering the maintenance decisions or maintenance optimizations based on the degradation models of the known system. To address this, we propose PMMI 4.0, a Predictive Maintenance Model for Industry 4.0, which utilizes a newly proposed solution PMS4MMC for supporting an optimized maintenance schedule plan for multiple machine components driven by a data-driven LSTM model for RUL (remaining useful life) estimation. The effectiveness of the proposed solution is demonstrated using a real-world industrial case with related data. The results showed the validity and applicability of this work.

## Introduction

Modern collaborative industry is moving toward applying the Industry 4.0 concept for achieving effective smart solutions (Zezulka et al., 2016; Thoben et al., 2017; Sang et al., 2020). Industry 4.0 supports the flexibility required for collaborative networks by the application of advanced technologies. The Internet of things (IoT), cyber–physical systems (CPSs), big data analytics, cloud computing, etc., are used for operating intelligent machines and processes in the collaborative context (Zezulka et al., 2016; Thoben et al., 2017; Koren et al., 2018). In Industry 4.0 focusing on the manufacturing context, business processes are executed across different factories and enterprises. This enables the collaborative chain to manage the production life cycle and demands effectively (Xu et al., 2020) as well as providing opportunities for supporting data-driven predictive maintenance within one organization as well as cross organizations (Sang et al., 2020a).

Predictive maintenance aids in the effective management of industrial assets. It provides a diagnosis of faults in related different machinery, deficient processes, and detailed inspection of the detection using various analyses (Mobley 2002; Sang et al., 2020b). Predictive maintenance involves two key components: inquisition of knowledge through analytics, i.e., prediction and detection of machine tools, and decision-supported schedule plan for the required maintenance task to be completed. Maintenance can be done in different ways, from conventional approaches such as corrective, preventive, to predictive approaches (Mobley 2002). The former relies on an expert knowledge, actual system degradation model and acts on a failure occurrence which is costly and unpredicted (Mobley 2002). The latter focuses on a data-driven approach utilizing various data produced by machine equipment tools, factory operation, and other information processing systems (Mobley 2002; Sang et al., 2020a).

Due to the emergence of Industry 4.0, the complexity of machine equipment involved in the modern collaborative industry has rapidly increased. Any failure of the machine equipment tool may have significant impacts such as downtimes (an estimation of $50 billion per year for the global industry by unplanned downtimes) (Deloitte 2021), and maintenance is key to operating the machine equipment effectively (Mobley 2002). Therefore, it is essential to have an effective strategy that efficiently coordinates the failure prediction or detection and maintenance schedule to ensure optimized operation and productivity. Industry 4.0–driven manufacturing systems are complex systems of strongly interconnected machines or devices that interact and collaborate for business processes toward common goals (Zezulka et al., 2016; Thoben et al., 2017). Hence, any maintenance activity to be performed in such complex systems should consider not only a single component but also the dependencies of various machine components involved. Therefore, a concrete maintenance strategy is essential for operation in an effective manner in a manufacturing system.

Industry 4.0 essentially simplifies the complexity of manufacturing collaboration by providing a consistent view of understanding the complex systems, processes, and collaborations. It facilitates different enterprises the ability to work with different collaborative partners with their systems or devices with required data using advanced technologies such as the IoT, cloud, and CPSs. In this context, the interactions facilitated by data exchange produce a large amount of data, not just from the collective system processes but also from the related environment where the processes are being executed. This poses several challenges to the design and implementation of predictive maintenance: data acquisition, data processing, and process interaction.

First, predictive maintenance requires *acquiring data* from different multiple sources to produce useful information and insights for analytics. Data come from various sources with different forms; hence, they must be handled properly. Second, existing *data processing* and tools are unable to process such data, i.e., big data, by traditional data processing methods and tools, and hence, new methods and advanced technologies supporting big data analytics, deep learning, etc., are required. Third, Industry 4.0 manufacturing is complex and *involves* different collaborative processes, systems, and partners. A flexible model is essential to allow for easy and interoperable integration of different processes and components for operating effective predictive maintenance.

Lee et al. proposed the architecture for designing smart manufacturing systems (Lee et al., 2015b). It involves five levels of steps which are done in a sequential manner. The approach may not be flexible for the modern industry to deal with dynamic changes and demands. Chiu et al. presented predictive maintenance focusing on a manufacturing cell, which mostly deals with monitoring the equipment tools (Chiu et al., 2017). Sang et al. offered the aspect of FIWARE predictive maintenance with some related functions (Sang et al., 2020a; 2020b, 2020). Furthermore, a semantic cloud framework for predictive maintenance was proposed by Schmidt et al. (2017). Their approach was based on domain ontology and was derived from challenges such as different domain data existing for predictive maintenance. Thus, this approach mainly deals with the aspect of data collection and analysis for predictive maintenance and however failed to address the challenges of flexibility, a significant aspect of operating Industry 4.0 predictive maintenance. This paper explores a way of supporting a predictive maintenance model in the context of Industry 4.0. This paper contributes to

a. investigating the Industry 4.0 predictive maintenance model for a series of machines within a product line for flexible manufacturing,
b. presenting a decision support method for scheduling predictive maintenance activities, which is utilized then by the predictive maintenance model, and
c. using the proposed predictive maintenance model and predictive maintenance schedule to apply to an industrial manufacturing case for verification.

To present the model, *Related Work* reviews related work. The results of this are used in *Predictive Maintenance for Industry 4.0* to underpin the predictive model for maintenance and maintenance scheduling optimization. This model is then used in *Predictive Maintenance Schedule for Multiple Machines and Components* as the basis of the proposed predictive maintenance schedule for multiple machine components. This model is evaluated in *FIRST Flexible Manufacturing Case* using a flexible manufacturing case derived from the FIRST project. The results of the case are discussed in *Discussion* to conclude in *Conclusion and Future Work*.

## Related Work

Advanced Industry 4.0 manufacturing systems are complex and involve advanced operating machines, smart sensors, and robots running on the shop floor as well as a network of collaborations including collaborative business processes, e.g., different systems (Zezulka et al., 2016; Thoben et al., 2017). The physical manufacturing machine equipment and systems of the manufacturing factory are expensive (Becker and Wagner 2016), and the state such as the condition, health, and operation of those machines can have a huge impact on the manufacturing chain. Any failure of the machine equipment tools can easily lead to undesired costs and disruptions such as delay and dispute in the value-added processes of the enterprises, partners, etc., due to the collaborative nature of production systems (Weber et al., 2016; Sang et al., 2020; Deloitte 2021). Essentially, any unexpected failure or inefficient process of manufacturing machine equipment may result in unintended downtimes and costs for an entire production line (Mobley 2002; Sang et al., 2020a; Deloitte 2021).

In the context of Industry 4.0 manufacturing, most literature studies in predictive maintenance can generally be distinguished into two aspects: one that focuses on big data analytics–enabled predictive models such as remaining useful life and the other that lies in maintenance scheduling optimization. In the next section, we review the related work for the predictive model for maintenance and maintenance scheduling optimization.

### Predictive Model for Maintenance

In the context of industrial maintenance management, prognostics and health management (PHM) is essential for reducing maintenance costs (Mobley 2002). In the context of PHM, remaining useful life (RUL) prediction is a key component and hence has attracted the attention of the research community due to its capability of determining the maintenance time. The RUL of machine equipment can be described as the time period between the present and the end of the useful life (Tobon-Mejia et al., 2012a; Sang et al., 2020a). RUL prognostics can be done in different approaches including model-based, data-driven, and fusion prognostics (Tobon-Mejia et al., 2012b; Sang et al., 2020a). The model-based methods solely rely on the degradation model of physical structure to the prognostic equipment health state. Therefore, they are not efficient in dealing with constraints such as a complex equipment structure (Tobon-Mejia et al., 2012a). To overcome this, data-driven approaches use various data such as sensor measurement, operational, to learn RUL prediction without the knowledge of physical structure and degradation. An alternative approach, fusion, can be considered in a way that both model-based and data-driven methods are used. However, it is still problematic for dealing with the physical structure which tends to be undiscovered intricacy. As such, data-driven methods have been proven to be effective in predicting RUL (Tobon-Mejia et al., 2012b).

Over the years, prediction methods based on sequence learning are the focus of data-driven research for RUL prognostics due to the intrinsic nature of the time series (Zheng et al., 2017). Furthermore, several machine learning approaches were proposed for prognostics models. The approaches mostly analyzed sensor time series data and discovered the associated patterns with a prognostics task (Srivastava and Han, 2016). These approaches offer an effective solution to the manufacturers (Mobley 2002; Tobon-Mejia et al., 2012a). The utilized techniques for prediction models include auto-regressive integrated moving average–based (ARIMA) models (Wu et al., 2007), hidden Markov models (HMMs) (Baruah and Chinnam 2005), support vector regression (SVR) models (Benkedjouh et al., 2013), artificial neural networks (ANNs) (Arnaiz-González et al., 2016), and random forest (RF) regression (Wu et al., 2017).

However, the demands of advanced prediction make it impossible for the traditional data-driven methods to handle the data complexity and growth (Mourtzis et al., 2016; Sang et al., 2020). Deep learning–based models have recently received great attraction as they offer several benefits such as better performance of RUL prognostics, i.e., high prognostics accuracy and automatic feature extraction (Wang et al., 2016b). In the context of RUL estimation, the convolutional neural network (CNN) is predominately used for the acquisition of high-level spatial features from sensor signal data (Ren et al., 2018; Mourtzis et al., 2020a). Moreover, long short-term memory (LSTM) neural networks are specifically adopted for extracting sensor temporal information (Zheng et al., 2017). In this instance, only temporal characteristics are considered for a single deep learning model. Alternatively, a combination of CNN and LSTM was proposed by Wang et al., 2016a; Ullah et al., 2017; Zhao et al., 2017. A framework for automatic generation of augmented reality based on the CNN is proposed for assisting maintenance (Mourtzis et al., 2020b). These approaches mostly focus on natural language processing, speech processing, video processing, etc. Moreover, the addition of a health indicator leads to accurate prediction results (Lei et al., 2018). Thus, more features can help describe equipment degradation for RUL prognostics.

Overall, most studies focus on the application and optimization of the predictive models (mostly public datasets and limited in the industrial manufacturing context) but do not consider decision support such as maintenance scheduling optimization using the predictive models in the context of Industry 4.0.

#### Long Short-Term Memory Network

The long short-term memory (LSTM) network is a type of RNN for sequence learning tasks (Hochreiter and Schmidhuber 1997). It can learn over long time sequences without compromising memory. For system prognostics, LSTM supports looking back the historical system states, i.e., degradation and tracking the states for RUL prediction (Hochreiter and Schmidhuber 1997; Zheng et al., 2017). This makes LSTM an effective approach in dealing with sequential sensor or time series data, and hence, it fits well for predictive models utilizing the data-driven approach for machine condition and RUL. In our PMMI 4.0, the LSTM RUL model is based on a hybrid approach, i.e., different layers of combination of networks are explored to handle both machine operation (sensor) data and condition data, e.g., status of the machine state, and the model drives the predictive maintenance schedule plan in the context of Industry 4.0.

To perform RUL prediction for prognostics, LSTM models can be constructed based on regression or classification. For regression, a piece-wise linear (PWL) is usually used for RUL target function (Zheng et al., 2017), and the result accuracy is mainly associated with the prediction horizon. However, defining the RUL maximum value is not trivial, and especially, an incorrect value can lead to an incorrect decision when using the predicted RUL value at the first stage of the system lifetime. On the contrary, classification techniques can be used without the PWL assumption, and the probabilities of the system failure into different time intervals can be achieved. This also means that different classes of learning represent the probability of the system RUL.

To demonstrate the concept, let us assume that sensors *s* are used for monitoring machines *M* of the same type during operation. The monitoring data for each machine component *d*, *d* = 1, … *M*, and the lifetime *Lt* can be expressed in a matrix form, *Md* = [*m1, … , mLt*]. To drive the learning of the model in the training stage, the sensor measurement sequences *Md*, *d* = 1, … , *N*, are taken as inputs by the LSTM network to determine the true RUL of the time window. To determine the RUL with each time window, the time step *t* and the constructed LSTM model take the vector of sensor measurements as input data and output the probable RUL.

To train the LSTM network, preprocessed data from multiple sources are necessary. This can be done in different ways such as normalization, data labeling, and formalization to meet the requirements of the LSTM model. In the context of normalization, input data are prepared and gathered from multiple sources. These data normally come with different ranges of values. For training the model such as LSTM, it is necessary to normalize every feature value by its mean and variance, and this leads to all features being within the same range, i.e., between zero and one. (Goodfellow et al., 2016; Zheng et al., 2017).

Data labeling is necessary for model training, either classification or regression (Goodfellow et al., 2016; Zheng et al., 2017). For RUL prognostics, data labeling can be processed on the operation engineers for the relevant time windows and required information, e.g. maintenance and production activities. In this sense, a maintenance engineer might require information of system failure in two different time windows, and data labeling then can be performed in two classes or time windows (e.g., w1, w2) using technique such as piece-wise linear. Moreover, the classes or time windows can be processed or extended as per different needs.

In the context of formalization, training models require the data input to fit into the corresponding model requirement. Regarding the LSTM model, a 3D tensor as the input layer is required for training the models and making predictions. The format of LSTM input data follows a three-dimensional array, sample (s), time step (t), and feature (f) (Goodfellow et al., 2016; Zheng et al., 2017). For the number of features f, different features can be obtained by extracting a sensor output using various methods, or sensor signals, i.e., one sensor for one feature, can be directly fed as input to the LSTM model (Wang et al., 2016a). The time step (t) specifies the number of time steps looked back by the LSTM network during model fitting and predictions.

To evaluate the model performance, different techniques depending on the learning methods, i.e., classification or regression, can be utilized. For instance, the technique such as the root mean square error (RMSE) is usually applied for regression approaches, whereas the method such as the confusion matrix is often used for classifications. In the case of RUL estimation, the RMSE is widely used as an evaluation technique in conjunction with a score function for measuring the quality of the models. The RMSE gives equal penalty weights to the model when the estimated RUL is different, i.e., smaller or larger than the true RUL (Goodfellow et al., 2016; Zheng et al., 2017).

### Maintenance Scheduling Optimization

Over time, the state, i.e., condition and health of factory machines, is hampered by the usage and age. And this eventually will cause a machine failure or deficient operation if no maintenance action is taken. Ultimately, machine equipment failures impact the entire manufacturing operation and can result in undesired downtimes and costs for the manufacturing chain. This impact, the corresponding downtime and cost of maintenance, can also be triggered by the excessive or unnecessary maintenance caused by the machine failure (Mobley 2002; Deloitte 2021).

Industry 4.0–driven manufacturing systems are complex systems of strongly interconnected machines or devices that interact and collaborate for business processes toward common goals (Zezulka et al., 2016; Thoben et al., 2017; Koren et al., 2018). Hence, any maintenance activity to be performed in such complex systems should consider not only a single component but also the dependencies of various machine components involved. Therefore, a concrete maintenance strategy is essential for operation in an effective manner in a manufacturing system.

Scheduling problems including maintenance activities may generally be represented by two common groups: one that focuses on pre-defined or fixed constraints such as duration of the activity (times including the start and end of the activity) and the other that focuses on more coordinated approaches that deal with conducting the process of maintenance activity and job simultaneously (Pinedo 2016). Traditionally, systems such as manufacturing execution systems (MESs) are used for the scheduling of manufacturing operations. This however cannot meet the demands of increased flexibility and scalability in dealing with diverse systems in a collaborative environment. This means that as manufacturing paradigms are embracing toward the concept of Industry 4.0 such as cyber–physical systems, new flexible approaches are needed for managing the manufacturing process efficiently (Zezulka et al., 2016; Thoben et al., 2017).

In the Industry 4.0 manufacturing context, maintenance is challenging as it associates with various linked systems and machine equipment, e.g., CPSs, IoT, robots, and CNC machines. The research community has explored different aspects of maintenance. In the case of traditional maintenance, single-component systems were mainly explored (Wang 2002; Chan and Asgarpoor 2006). These approaches mostly consider a single machine or component and overlook the related machines/components. Thus, the aspect of multi-component system maintenance becomes the focus of various works (Dekker et al., 1997; Nicolai and Dekker 2007; Van Horenbeek and Pintelon 2013). In this context, machine equipment with more than one component was considered. Also, additional considerations such as economic cost related to downtime and machine are recognized by Dekker et al. (1997) for cost savings.

Furthermore, Mourtzis et al. (2017) proposed an integrated system, under the concept of Industry 4.0, which utilizes data gathered from the monitored equipment and adjusts the maintenance schedule upon timeslot availability. Senra et al. (2017) proposed an approach that considers available equipment with support technicians, as well as the related processing times for the schedule process. The proposed approach was illustrated with a case study that however lacks equipment monitoring for analytics. Another method for the decision-making tool was proposed for production maintenance synchronization (Levrat et al., 2008). It utilizes multiple criteria such as product performance and component reliability for producing an optimal scheduling plan. In the context of flexible job shop scheduling, Zheng et al. (2013) focused on a scheduling problem incorporating a condition-based maintenance approach for providing the optimal solution. However, it lacks the consideration of the applicability of different types of machines and the overall schedule for producing a new maintenance schedule.

To respond to the maintenance schedule problem of complex systems in Industry 4.0, an optimized solution is required. This needs the consideration of complex systems, with multiple machine components involved in a typical domain such as manufacturing. Furthermore, data-driven approaches are effective in dealing with maintenance management, compared with the traditional corrective and preventive techniques which cannot deal with the complexity and demands of the Industry 4.0 domain (Mobley 2002; Tobon-Mejia et al., 2012b; Sang et al., 2020a). Essentially, the data-driven maintenance schedule plan utilizes predictive models derived from the historical, operational, and condition data of the machine equipment. Predictive models such as RUL detection are developed using machine learning or deep learning techniques for assisting better maintenance (Mobley 2002; Pinedo 2016; Sang et al., 2020a).

In addition to a data-driven predictive approach, an optimized maintenance schedule should consider the cost associated with maintenance activity, instead of solely focusing on the scheduled task. This includes the cost of the duration of the maintenance task. Essentially, the objective is to minimize the cost by considering multiple machine components with associated maintenance tasks, operation or downtime time, and the optimal time for maintenance to be deployed (Mobley 2002; Pinedo 2016; Sang et al., 2020a).

In our proposed PMS4MMC, what we have thus considered is utilizing a predictive model and decision-supported maintenance schedule plan for multiple machines/components involved in complex manufacturing, specifically in the context of Industry 4.0 predictive maintenance.

## Predictive Maintenance for Industry 4.0

Industry 4.0 operates with advanced technologies such as the Internet of things (IoT), cyber–physical systems, smart sensor devices, cloud, and big data analytics. In general, operations in a manufacturing system are facilitated by a digital platform, smart machines, and networks that are collaboratively linked. Industry 4.0 enabled technologies to provide more information for operating an advanced predictive maintenance solution. Smart machines and machines that are networked can support the better data-driven prediction of the RULs of the individual machines or related components. For making the decision on when to do maintenance in the scope of (different constraints) different constraints (e.g., availability of engineers, hardware), the information of the manufacturing and related business processes is facilitated to optimize results by satisfying multiple criteria.

In this section, the architecture of predictive maintenance for Industry 4.0 is presented in *Architecture of Predictive Maintenance for Industry 4.0*. To support predictive maintenance, the related data types and data model are introduced in *Data Types and Data Model for Predictive Maintenance for Industry 4.0*. An overall predictive maintenance process is described in *Predictive Maintenance Process and Predictive Maintenance Model for Industry 4.0* (also see *Section Predictive Maintenance Process and Predictive Maintenance Model for Industry 4.0*) which includes data acquisition in *Data Acquisition for Predictive Maintenance*, data process and prediction in *Data Process and Prediction*, and maintenance decision support in *Decision-Supported Maintenance*. One of the main contributions is the predictive maintenance model for Industry 4.0 (PMMI 4.0) provided in *Predictive Model for Maintenance*.

### Architecture of Predictive Maintenance for Industry 4.0

There are different implementation platforms for supporting Industry 4.0. FIWARE is an open-source framework for industrial smart solutions, and flexibility is facilitated by the ability to easily integrate different components for different needs in a modular fashion (Catalogue 2021). FIWARE is adopted in this research for several reasons such as flexibility, interoperability, and supporting big data analytics, by supporting an open and industrial standard data model allowing for the ease integration of different IoT smart devices, systems, etc.

**FIGURE 1:**
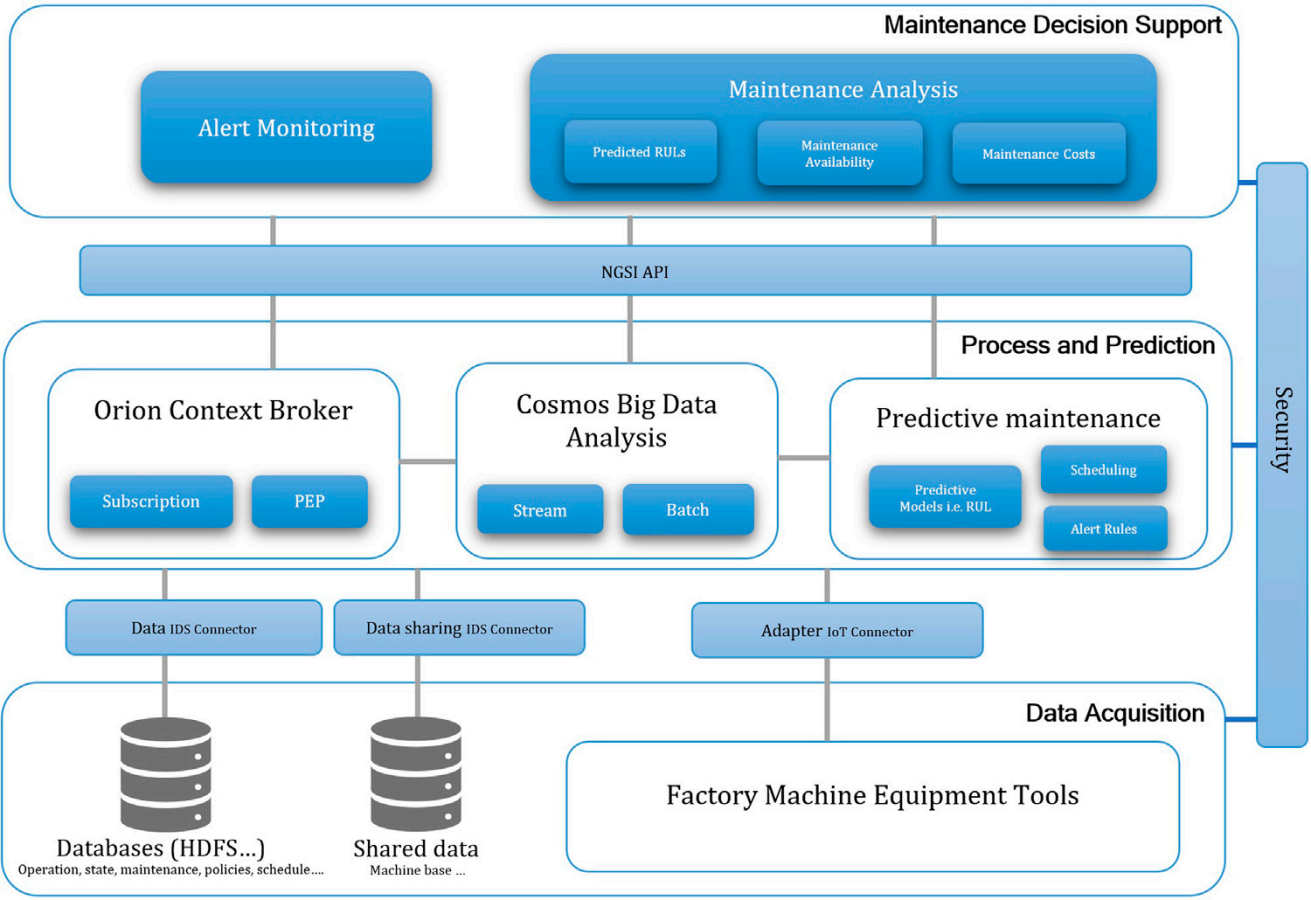
PMMI 4.0 architecture based on FIWARE.

Industry 4.0 operates with several different machines, i.e., robots, IoT devices, and CNC machines, and the nature of dynamic data can be extremely frequent and highly voluminous. The designed architecture requires to support interactions among the different machines/components for predictive maintenance by the characterization of end-to-end integration and processes with different security needs such as identity and privacy. The PMMI 4.0 architecture based on FIWARE is thus depicted in Figure 1. At the lower level, the data are collected from different adapters or databases. At the middle level, the Orion Context Broker and Cosmos Big Data Analytics are provided by the FIWARE architecture. We add the predictive maintenance module for holding predictive maintenance functions. At the top level, different visualization of maintenance analysis results provides an interface to monitor or interactively configure the maintenance schedule.

### Data Types and Data Model for Predictive Maintenance for Industry 4.0

Generally, the different types of data required for predictive maintenance include the following:

⁃ Operation data: diverse business factory operation/condition data generated by a variety of IoT, CPSs, devices, machine equipment tools, etc.
⁃ Defect data: historical data about events that occurred regarding fault or breakdown to the asset including the type of fault or breakdown, reason, and timestamped.
⁃ Maintenance/repair data: historical maintenance data of the assets including replacement, executed tasks.
⁃ Machine data: historical operational data of the assets including the status of the machine, state information such as machine critical parameters, specification, uptime, downtime, and an alert indicator such as oil low.
⁃ Manufacturer’s data: measurements and control data (base data) from the manufacturer of the asset.

**FIGURE 2:**
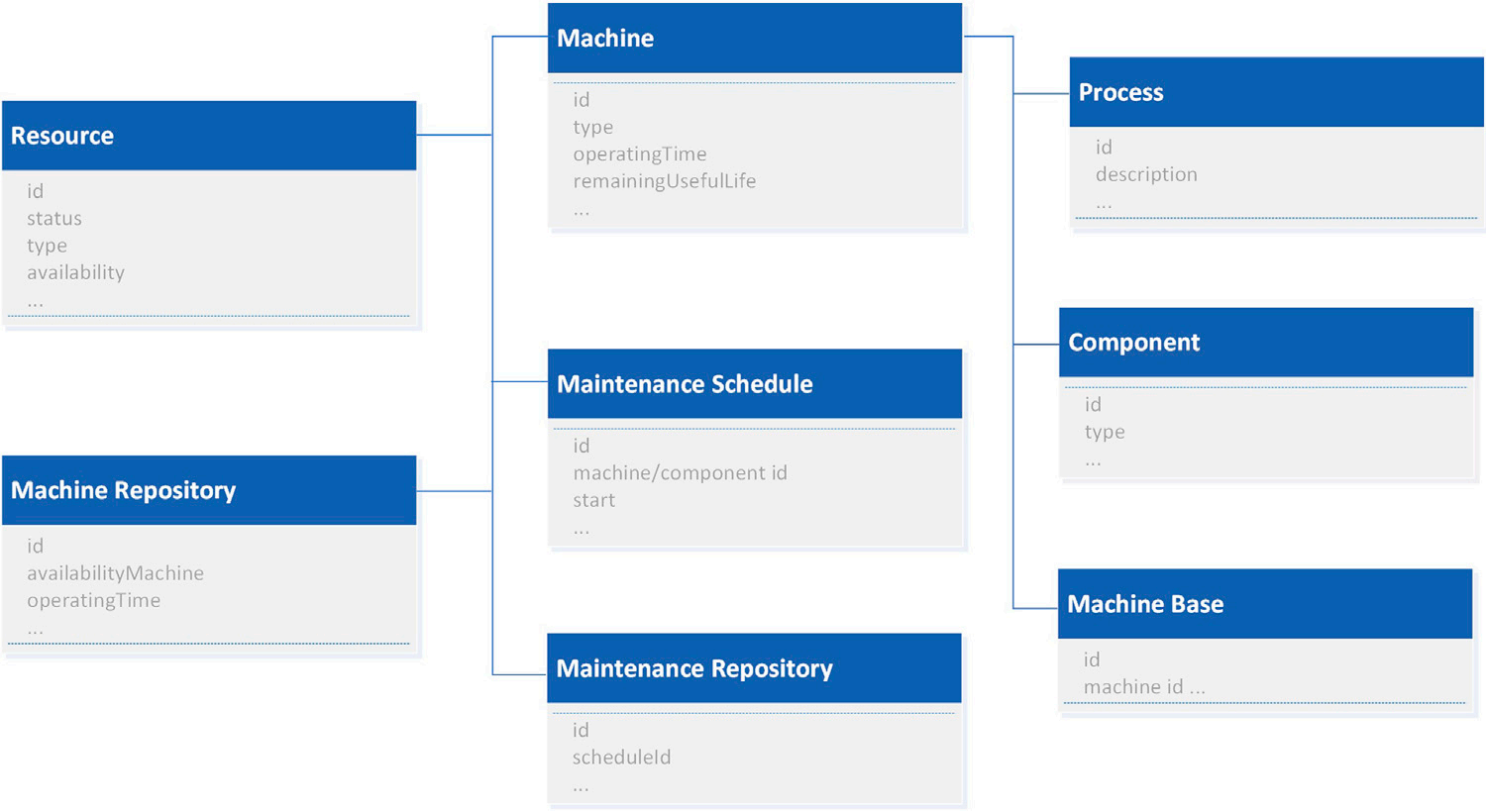
Sample data model for predictive maintenance for Industry 4.0.

A data model captures data from the resources and their dependencies which could lead to an effective predictive maintenance solution and making maintenance data available for decision-makers. The data model for predictive maintenance for Industry 4.0 is constructed in Figure 2. It depicts the resource, machine repository, maintenance repository, maintenance schedule, machine, component, process, and machine base. The *resource* stores data about factory machine equipment tools including their dependencies such as the configuration/location/type of the machine component and maintenance engineer. The *maintenance repository* stores related maintenance data including existing maintenance schedules. *Maintenance scheduling* is facilitated by the data model for maintenance-, machine-, or resource-related data. The *machine* stores data about the individual machine equipment or tool, type, etc., where it can also have a *component*(s). The *machine base* refers to the machine-specific data such as specification and configuration for each machine from the manufacturer. The *process* stores the specification of the factory process for the machine equipment tools. These data are made available for the decision-supported maintenance in assisting maintenance decisions. The current model depicts a sample model, and it can be extended as required.

### Predictive Maintenance Process and Predictive Maintenance Model for Industry 4.0

**FIGURE 3:**
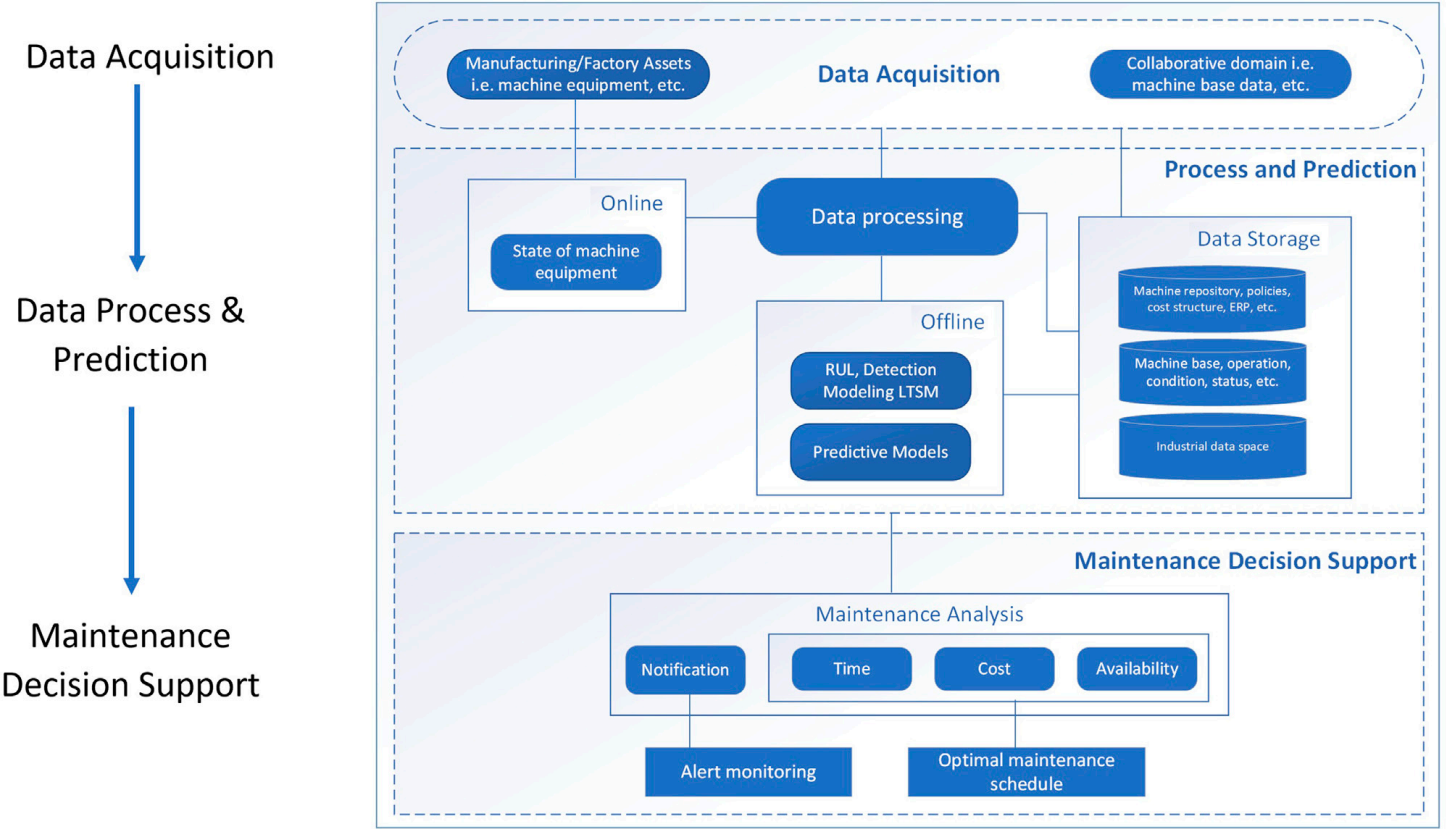
Overall predictive maintenance process and framework.

In this section, an overall predictive maintenance process is described in Figure 3A. Data acquisition is discussed in *Data Acquisition for Predictive Maintenance*, which is critical to operating the maintenance operation efficiently. The second step of predictive maintenance is the data process and prediction. The collected data from entities or resources are processed for reducing a significant impact on the manufacturing chain in case of their failure. We propose a predictive maintenance model for Industry 4.0 (PMMI 4.0) in *Data Process and Prediction*, which can predict the remaining useful life (RUL) of machines/components. The second step provides a base for supporting maintenance decisions. The third step of the process is maintenance decision support. This step covers the general aspect of maintenance assisting an operator, i.e., maintenance engineer, to act on an event prompting to perform a certain maintenance task. Different user interfaces or dashboards are also included to aid the users in interacting with the predictive maintenance platform. A detailed discussion of the supporting maintenance decision is given in *Decision-Supported Maintenance*.

#### Data Acquisition for Predictive Maintenance

Data acquisition for predictive maintenance concerns with collecting and processing data from enterprise assets. The enterprise asset refers to the entity or resource which is critical to operating the factory operation at peak, efficiency, and utilization to realize that its failure can have a significant impact on the manufacturing chain. These assets could include production machines, equipment, tools, industrial devices, and factory-related resources.

In a flexible manufacturing setting, various data are collected during operation. These collected data generally include event, condition, and operation data. Operation data may include capturing data of certain process, whereas event data may be collecting data about the assets, i.e., machine equipment, regarding what happened to the asset and what maintenance was applied to it. Condition data may involve collecting data about the general condition, i.e., health, and measurements of the asset. Moreover, using different sensors such as accelerometers and rain sensors, different signal data such as vibrations, temperature, pressure, humidity, and climate can be acquired as part of the general data collection, i.e., event or condition. In addition to acquiring various kinds of data from manufacturing machine equipment, tools, and systems, data from other collaborative partners are also being processed.

Generally, data collection is an online processing activity in the collaborative manufacturing chain. For predictive maintenance analytics, data must be captured from the different operating machine equipment tools of a flexible manufacturing product line. The online operating data acquisition allows for synchronous data operation, i.e., data to be collected from the factory and its product line. Moreover, real-time data collection should reflect the machines’ operating conditions. The collected data are stored in different data storage such as Hadoop HDFS, NoSQL, and relational database for different needs such as streaming data for specific NoSQL, staging processing, and multi-dimensional series or time series for analytics.

Using PMMI 4.0 supported for the Industry 4.0 framework in Figure 3B, different source data could be collected from sensors, smart machines, IoT, and different places such as Hadoop HDFS, NoSQL, and IDS. On data acquisition of the PMMI 4.0 framework in Figure 3B, the *manufacturing assets*, i.e., factory machine equipment tools, operate and connect with the middleware Orion Context Broker (the middle layer in Figure 2), related processes, and data storage *via* different associated FIWARE’s adapters (the low layer in Figure 2). The middleware context broker represents the communication mechanism with different adapters and the related data sources and storage required for the platform. FIWARE Orion Context Broker acts as the middleware to facilitate the life cycle of the context information including registrations, updates, subscriptions, and queries using NGSI REST API and PEP Proxy for interaction and security enforcement and IDS connectors for data access and control. For the aspect of security concerns such as privacy and encryption, Keyrock is applied with IDS connectors.

#### Data Process and Prediction

The process and prediction refer to the general processing and modeling required for building a predictive model or acquisition information for analytics.

Data processing involves the general processes conducted to generate information from the huge amount of data collected. Data from data acquisition are stored in different *data storage* such as Hadoop HDFS, NoSQL, and relational database for different needs such as streaming data for specific NoSQL, staging processing, and multi-dimensional series or time series for analytics. In addition to manufacturing factory operation data including related machine equipment tool data, the manufacturing industry has data in related business information systems, such as ERP and logistics. Also, collaborative data such as product design and machine-based data are facilitated by Industrial Data Space (IDS) or other collaborative business systems.

Raw data must be transformed into actionable knowledge for decision-making. Generally, methods such as data cleaning, preprocessing, and reduction are performed for various analyses and modeling. For data cleaning such as resolving missing values, the format can be performed, while data processing such as resolving inconsistent or redundant data can be done as part of preprocessing. Data reduction generally concerns with processing such as transforming data into meaningful and simplified forms by means of feature or case collection.

In the context of the PMMI 4.0 framework as depicted in Figure 3B, data processing generally concerns with both *real-time (online)* and *offline data*. For the *online aspect*, the operating condition of the machine equipment tool of the factory system is considered for real-time monitoring and notification. Generally, real-time data about the condition, i.e., health, and measurement of the factory machine equipment tools are processed and stored in a database, facilitating the functional aspect of notification and monitoring. The *offline aspect* generally concerns with the historical data and enterprise data collected from various processes and operations. These data are ultimately used for developing various analytics solutions such as predictions. In the data acquisition process of predicting maintenance, data are typically collected from multiple devices including sensor-enabled ones. This requires the integration between the lower level of predictive maintenance and the information level *via* adapters such as FIWARE IoT adapters. In this process, both real-time and batch processes are supported by different methods such as real-time signal processing and vibration. In the preprocessing stage, data collected are cleaned, prepared, and formatted as required for building specific predictive models or general analytical functions (Sang et al., 2016, 2017).

To facilitate the capability of advanced big data analytics for the PMMI 4.0 architecture in Figure 2, FIWARE’s Cosmos Generic Enabler, which supports big data analytics including streaming and batch data processing, along with different available IoT and device adapters, is adopted. The Cosmos GE enables big data analysis of both batch and stream data. It includes a Hadoop engine, an authentication generator based on NGSI API, and a connector to FIWARE’s context broker (FIWARE 2021). Data can be collated into Cosmos Big Data GE *via* a shell which is the Hadoop command line interface or by injecting into HDFS using Telefonica’s SSH File Transfer Protocol (SFTP) server. Cosmos Big Data GE provides an interface for SFTP which facilitates transferring files into the platform (FIWARE 2021; Hadoop 2021). Then, the results of MapReduce can be consumed *via* HDFS for access, i.e., applications or users. Orion Context Broker is the middleware that facilitates the access of other applications or users. Different big data–enabled components such as STH Comet/Cygnus for data persistence can be integrated based on different requirements, i.e., real-time and batch data processing, allowing the ability to integrate with different functions as a plug-in/plug-out option.

##### Predictive Model for Maintenance

There are various examples of predictive maintenance models such as RUL and wear detection (for wear failure and degradation). These tools, need to be trained and evaluated before deployment. The models can then be integrated with the platform for failure/degradation prediction/detection. Maintenance predictive models incorporated with related maintenance information provide a basis for determining the predictive maintenance schedule plans.

Remaining useful life (RUL) is adopted for PMMI 4.0 predictive maintenance since it is being recognized simply by being able to accurately estimate the end of life of a machine component (Si et al., 2011; Tobon-Mejia et al., 2012a; Babu et al., 2016; Zheng et al., 2017). In this sense, maintenance based on predictive RUL can facilitate better optimization such as in time acquisition of resources, e.g., spare parts and engineer, and ultimately effective scheduling. The difference between high accuracy and medium accuracy may also mean significant savings in cases, where complex multiple systems/components are maintained, and maintenance costs are high. Predicted RUL and its corresponding horizon can be used for determining performance parameters that can lead to predicting the failure time.

In the context of Industry 4.0, resource dependency such as machines/components in a product line is from one manufacturing organization to collaborative multiple organizations. The maintenance-related data of each machine/component, condition, etc., need to be captured. For traditional manufacturing organization, resource dependency may not be as critical as the Industry 4.0 collaborative aspect, since the traditional organization does not need coordination or data outside its own organization, and it can probably has its own capable resource. For an effective predictive maintenance, these resource dependencies must be considered, especially for scheduling (Sang et al., 2021). For developing the predictive RUL models, a similar type of machine equipment tool is required (Zheng et al., 2017).

**FIGURE 4:**
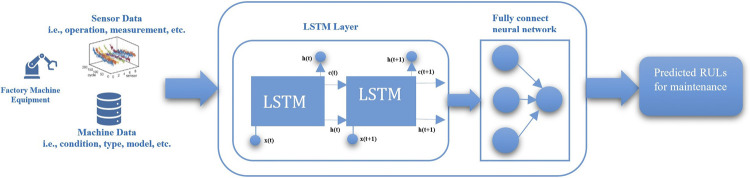
PMMI 4.0 predictive RUL model for maintenance.

For developing RUL predictive models, manufacturing machine/equipment operational and condition data are collected *via* an IoT sensor. These data are generally sequential sensor/time series data, and a long short-term memory (LSTM) network is effective in dealing with these data, compared with methods such as HMMs, ARIMA models, and RNNs (Bengio et al., 1994; Hochreiter and Schmidhuber 1997; Baruah and Chinnam 2005; Martens and Sutskever 2011). To support data-driven predictive maintenance, LSTM for a predictive RUL model is used for PMMI 4.0 (Sang et al., 2020a). In this work, a hybrid approach, i.e., different layers of combination of networks, is explored to handle both machine operation (sensor) data and condition data, e.g., status of the machine state. Zheng et al. (2017), Ren et al. (2018), Al-Dulaimi et al. (2019) use different LSTMs for the predictive RUL models which are not designed in the context of Industry 4.0. The model architecture is presented in Figure 4. A more detailed design of PMMI 4.0 can be found in Sang et al. (2020a).

In the context of training the LSTM RUL model, historical data of factory machine data, i.e., operation, condition, and maintenance data, can be used. The factory machine may include machine operation and condition data collected during factory operation, and the sample data used from the FIRST project are later described in *Maintenance Scenarios*.

The developed LSTM RUL model can then be deployed and consumed *via* NGSI API. It can then be set up for either online or offline use, depending on business requirements. Upon triggering the model, a list of potential machine components for corresponding RUL values can be made available. The RULs then can be used as inputs as well as related maintenance data for maintenance planning.

##### Maintenance Monitoring

One important aspect of maintenance is online (real-time) monitoring and notification regarding the critical condition of the production machine equipment tools. In this regard, these critical machine equipment tools are the maintenance assets. Typically, alert indicators and key state information such as specific configurations or parameter settings, oil, or pressure level of the maintenance items are based on the characteristics of each item acquiring maintenance. During factory operation, real-time data collected from the maintenance asset are processed for monitoring and determining a qualified notification.

In the *online (real-time) processing* described in Algorithm 1, several underlying machine equipment of manufacturing is considered. Operating these machine equipment tools derived real-time data, or the key state represents the corresponding state of each item. The threshold value of each item’s state is represented by the alert level of *N*. Each item alert *N* represents the alert indicator (normal, abnormal). For the executable maintenance task, the alert will be triggered by the threshold when either it is above the alert level or the indicator is abnormal. In this context, certain maintenance tasks such as minor adjustments are considered for automation. After the task is completed, the corresponding alert item *N* is set to normal. In the context of an unresolved problem, i.e., the task cannot be solved, an operator/technician will be attended, and the corresponding alert item *N* will then be updated as normal.

Algorithm 1

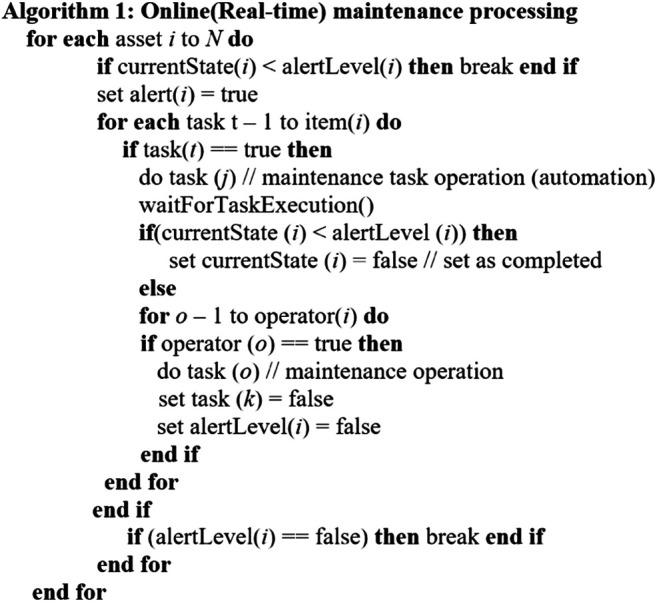

In the context of PMMI 4.0, different FIWARE’s components can be integrated as requirements. In this case, maintenance alert rules, e.g., detection of different thresholds such as failure, low-level oil, and temperature, can be configured as part of FIWARE’s complex event processing for real-time analytics, connecting with Cosmos Spark stream processing *via* Orion Context Broker. Based on the nature of the alert notification, the maintenance engineer can take appropriate actions.

### Decision-Supported Maintenance

The decision-supported maintenance generally concerns the user interfaces that facilitate user options for different applications including decision-supported maintenance analytics, i.e., schedule plan, decision-making, and other interfaces, visualizations for real-time monitoring, and alert notification.

**FIGURE 5:**
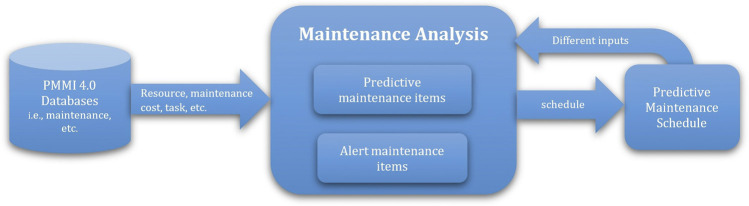
PMMI 4.0 maintenance analysis for decision-supported maintenance.

For the maintenance analysis, the outputs of the predictive RUL model that forecast the future RULs of machine components are considered inputs together with maintenance cost and resources, i.e., engineer and availability, to perform the optimization and produce the optimal maintenance schedule plan for assisting decision-making as illustrated in Figure 5. The analysis outcomes derived from the predictive models that forecast future machine conditions are utilized for assisting the decision-making process. Similarly, the alert maintenance items of maintenance monitoring can also be managed by a maintenance engineer.

To support the dynamic nature, i.e., new data such as machine operation/condition and maintenance, different inputs can also be used for handling new RUL values of machines components, e.g., for different RUL values or maintenance time, and then an optimal maintenance schedule plan can be created. The output can be consumed *via* FIWARE’s REST API. For maintenance analysis, the information derived from the predictive maintenance schedule and factory maintenance–related information available on the platform can be used against the operating machine equipment tools, and appropriate maintenance decisions, i.e., appropriate maintenance tasks and schedule plan, can subsequently be made. More detailed explanations of decision-supported maintenance are provided in *Predictive Maintenance Schedule for Multiple Machines and Components*.

## Predictive Maintenance Schedule for Multiple Machines and Components

Industry 4.0 predictive maintenance must consider the multiple machine components involved in factory operation. Moreover, separate maintenance for each of the multiple machine components at different times is highly expensive (Wildeman et al., 1997; Van Horenbeek et al., 2010; Van Horenbeek and Pintelon 2013). The resource availability of each machine component, the type of each maintenance, i.e., repair or replacement, and the setup cost of each maintenance, i.e., shutdown and up, can be daunting and costly. Hence, considering the resources while coordinating potential pending failures within a time window is much desired.

In this section, the approach for Industry 4.0 maintenance optimization is presented in *Approach for Industry 4.0 Maintenance Optimization* including key factor requirements for Industry 4.0. Adopting the approach along with identified factors, a data-driven predictive maintenance schedule driven by the predictive model supporting multiple machine components is proposed in *Proposed Predictive Maintenance Schedule for Industry 4.0 Multiple Machines and Components*. The proposed solution is implemented with PMMI 4.0 in *Predictive Maintenance With PMMI 4.0 and PMS4MMC*.

### Approach for Industry 4.0 Maintenance Optimization

Most existing studies focus on either a predictive model or maintenance optimization with one single machine and limited attempts of multiple machine components, especially in the context of Industry 4.0 (Dekker 1996; Wang 2002; Chan and Asgarpoor 2006; Dekker et al., 1997; Van Horenbeek and Pintelon 2013). These studies focused on maintenance aspects such as structure (structure of the machine), stochastic (machine failure degradation affecting other machines), and economic maintenance (opportunistic for grouping) optimizations that have been explored in the context of preventive or reactive maintenance. However, the aspect of predictive maintenance and Industry 4.0 is still overlooked. As such, we introduce the resource aspect for considering dependencies such as engineers to better meet the demands of Industry 4.0.

Handling the demands, i.e., highly collaborative complex systems and multiple machines of Industry 4.0, focusing on manufacturing is challenging (Thoben et al., 2017). From the requirements of Industry 4.0 in the literature studies (Lee et al., 2015a; Thoben et al., 2017; Sang et al., 2020a, 2020b; Sang et al., 2020; Zonta et al., 2020; Sang et al., 2021), several key factors need to be considered for an optimal maintenance schedule plan of PMS4MMC. This includes data-driven maintenance, i.e., predictive models such as RUL, multiple machine components, maintenance tasks, maintenance time, cost, and the resource aspect, i.e., availability status of each component and engineer.

**a. Data-driven maintenance**: This refers to the utilization of collected big data for building predictive models such as RUL that facilitate advanced detection of failures/degradation of factory assets. In this work, the predictive RUL is adopted. Traditional maintenance approaches such as preventive and reactive act upon failure events or routinely planned schedules. This results in undesired downtime as well as costs including maintenance cost which can cover engineer, downtime, etc. Predictive maintenance however is driven by predictive models derived from historical machine data, i.e., operation and condition. And this facilitates advanced detection of potential failures and enables timely pre-failure interventions. Thus, it provides an effective way of managing maintenance using the predictions as well as other maintenance information.

**b. Resource**: This refers to the general resources required for the maintenance, especially in the Industry 4.0 setting. In the context of existing approaches, the structure aspect generally focuses on the structure of one machine itself, the stochastic aspect relies on the degradation process of a machine, whereas the economic aspect focuses on cost saving by grouping maintenance activity. The aspect of resource however aims to enhance maintenance optimization for Industry 4.0 by considering resources such as maintenance operation of the machine equipment with associated components, processes, people such as the engineer and operator, and costs. Conducting a maintenance schedule plan and execution requires these resources as a whole system.

**c. Availability**: This refers to the status, i.e., availability of the above resources for the maintenance operation. Thus, resource availability is essential for conducting a maintenance schedule plan and execution for the whole system. It is important to get the information of the machine equipment which is required for maintenance but also to coordinate with other activities, processes, etc., to schedule an optimal maintenance task with minimal impact.

**d. Multiple machines**: In the context of Industry 4.0 focusing on manufacturing systems, various linked systems and machine equipment, i.e., CPSs, IoT, robots, CNC machines, of a highly collaborative network operate toward fulfilling a certain production goal. Any failure of the underlying machines can halt the whole manufacturing process. To reduce downtimes and costs and maintain optimized machine equipment tools, the consideration of different key machines’ components involved and operating in the manufacturing process is essential.

**e. Maintenance task:** This task regarding machine equipment can be varied from replacement of a component of machine equipment to minor or major repair of existing machine equipment. A typical corrective maintenance task can be fixing a failure of a component which can considerably be a more significant task, compared with a typical predictive maintenance task, whereas it might, in some cases, only need readjustment of settings, e.g., setting CNC machines to better cope with a potential failure or a certain production process. In the case of dependent maintenance, conducting a maintenance task can be increased by the required task accommodating the dependent machine component, e.g., stopping the machine, and restarting it in line with the maintained machine equipment.

**f. Maintenance time:** This can cover the time from preparation to conducting a maintenance task, the interval between stopping and restarting the machine, the time for a maintenance operator or engineer to conduct the maintenance task, and the actual time for the maintenance work of each machine component repair, replacement, readjustment, displacement, etc. The duration of maintenance can also depend on the condition status of the maintenance task, i.e., failure and worn. The sum of these variables can be considered the overall downtime of a production line, affecting the whole collaboration chain.

**g. Cost:** The most common optimization standards for preventive maintenance are based on cost minimization. The aim of cost minimization is to lower the overall maintenance expense. For a factory production line, the cost can range from sending a maintenance team to the site, stopping the production, to resetting the production environment. Thus, it is often economically beneficial to carry out maintenance actions of multiple components simultaneously. Regarding the maintenance cost, a typical fixed cost is incurred. To capitalize the maintenance fixed cost, it is desirable for a maintenance visit to conduct the maintenance activities for several components in one joint maintenance interval, rather than for a single component. This can lead to saving the overall maintenance cost for the whole system, compared with conducting maintenance tasks separately for each component at different time moments. This can be described as economic dependency (Dekker et al., 1997). Regarding the cost and related loss such as quality, preventive maintenance with a threshold could reduce the overall cost of corrective maintenance and quality loss.

Moreover, predictive maintenance scheduling deals with multiple inputs (i.e., the described factors) such as different RULs of different pending failure periods, maintenance associated with cost, and resource availability. Thus, efficiency is important. In this work, the maintenance schedule is considered dynamic, which means different input parameters can be adjusted or changed for different schedule plans. Different RUL values of a machine component or all can be amended as an input for some business reasons, e.g., different time windows may need to be considered for potentially not fulfilling orders.

### Proposed Predictive Maintenance Schedule for Industry 4.0 Multiple Machines and Components

**FIGURE 6:**
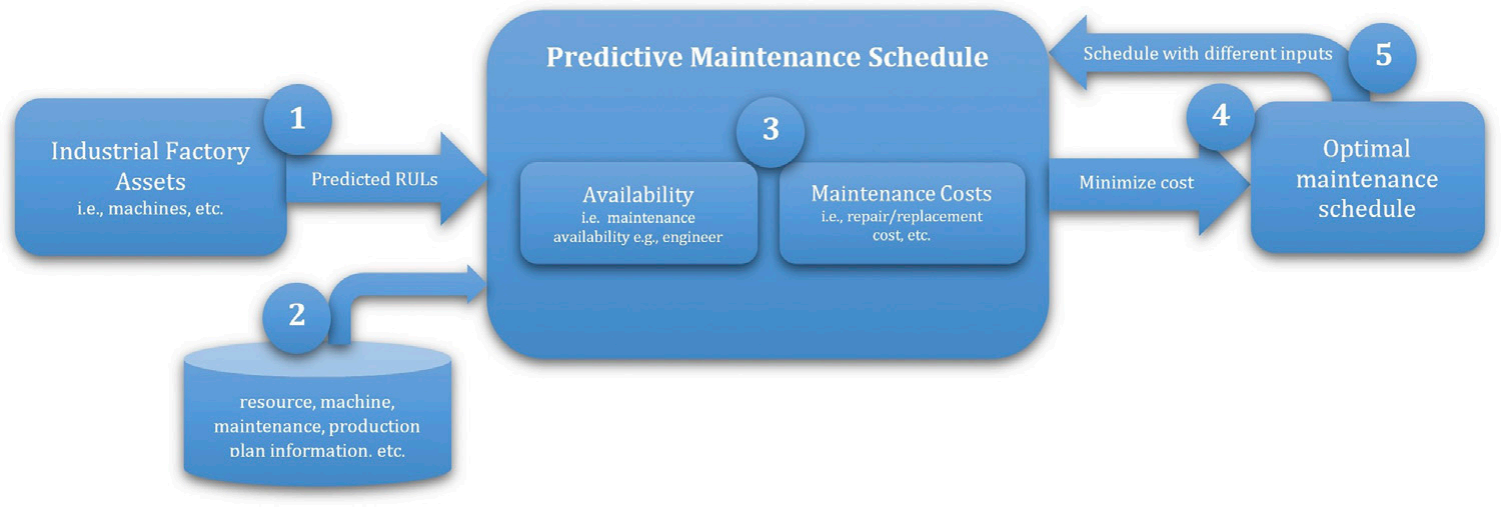
Overall predictive maintenance schedule procedure.

In general, predictive maintenance scheduling is seen as an optimization process, i.e., minimizing the cost that is driven by data-driven predictions, i.e., RULs from the predictive model and related data, i.e., maintenance, to assign the resources over time regarding the maintenance activities as illustrated in the overall predictive maintenance schedule procedure in Figure 6. Maintenance includes predictive RULs, multiple factory machine components, maintenance tasks, timestamps, and related costs. The No. 1 input of Figure 6 represents the result of “data-driven maintenance” listed in *Approach for Industry 4.0 Maintenance Optimization*. The No. 2 input of Figure 6 indicates the remaining list in *Proposed Predictive Maintenance Schedule for Industry 4.0 Multiple Machines and Components*. The No. 3 input “predictive maintenance schedule” in Figure 6 shows different schedule focuses such as maintenance availability and maintenance cost. It depends on which optimize factory(ies) is(are) selected by the user, for example, 4 in Figure 6 is selected as “minimize cost.” After the “optimal maintenance schedule,” based on the input of “minimize cost,” i.e., No. 5 in Figure 6, different predictive maintenance schedules will be provided.

The objective of data-driven predictive maintenance is to offer an optimal maintenance schedule plan driven by the RUL values of the corresponding machine components incorporated with related factory maintenance data, cost, task, and resource which minimizes the overall cost related to conducting the required maintenance and thereby reducing the downtime and cost. The goal of the maintenance task, i.e., short, medium, or long term, may become critical, depending on the degree of the task, time, and cost.

Considering the key factors described in *Approach for Industry 4.0 Maintenance Optimization*, the following algorithms are established. Algorithm 2 describes the overall *predictive maintenance schedule* which invokes related algorithms for executing the process. Algorithm 3 concerns with getting the maintenance assets, and Algorithm 4 deals with the maintenance cost aspect. Algorithm 5 concerns with maintenance time and availability of the required maintenance items, whereas Algorithm 6 deals with the aspect of availability.

Following the procedure (i.e., Figure 6) which utilizes Algorithms 2 **–** 6, the predictive machine schedule can be explained as follows:

1. First, machine sensor data, i.e., operation and condition, must be processed for the predictive RUL model as described in *Predictive Model for Maintenance*. This then produces the RUL model which can be deployed and run against potential maintenance machine components. Then, maintenance items with predictive RUL values are generated from the predictive RUL model. It provides pending maintenance items due for a future time window, e.g., 5 days, based on the RUL values. The predictive maintenance items with RUL values, e.g., a predictive remaining useful life value of 5 days (an example of this can be found in Figure 10B), drive the predictive maintenance schedule as described in Algorithm 2 *.*

Algorithm 2

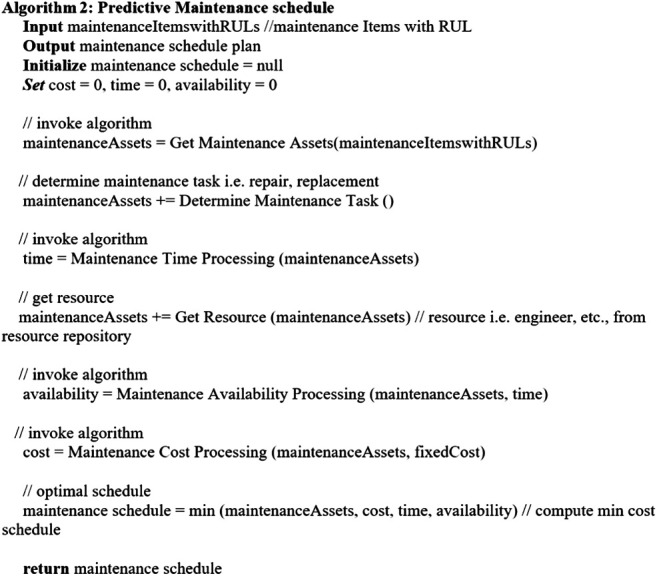

2. The input parameter of maintenance items with RUL is being processed to retrieve any corresponding pending machine or component items invoking Algorithm 3 *.* The machine repository is used for getting the corresponding multiple machine/components of the required maintenance items. This machine information is stored in the machine repository which is part of Figure 6 (i.e., No. 2).

Algorithm 3

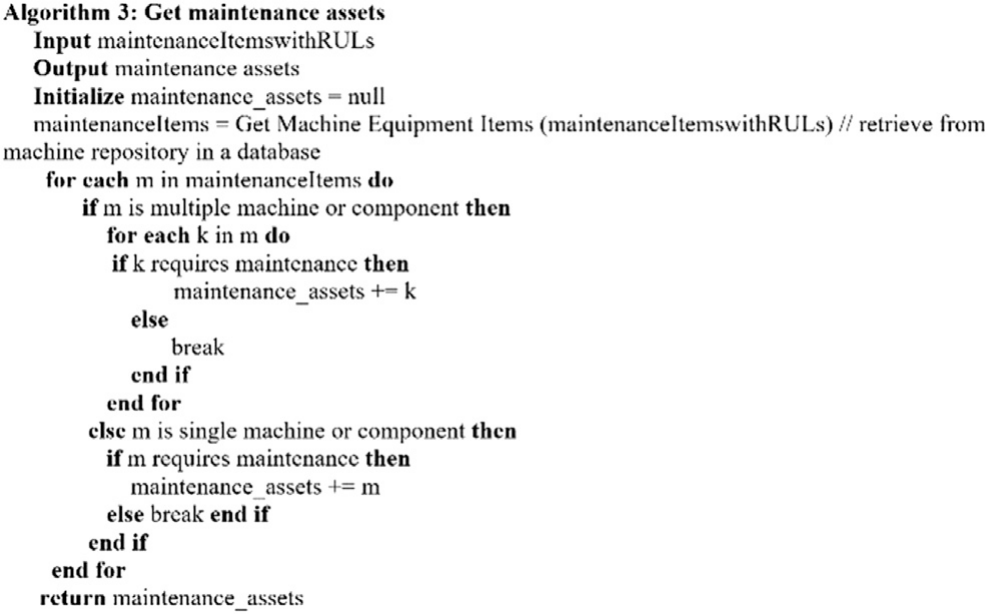

Algorithm 3 describes maintenance asset processing considering multiple machine components which require related maintenance. The machine repository is used for getting any outstanding multiple machine component maintenance for maintenance activity within the same time window period of the input maintenance RUL items. For the aspect of multiple machine components, each maintenance item is processed through its related machine components, and only maintenance-required items are considered.

3. Then, the next process, i.e., No 3 in Figure 6, proceeds. In this process, different algorithms are invoked and processed for the required maintenance.

First, the *maintenance task* is determined, based on the nature of the pending failure. The process can be varied from the replacement of a component of machine equipment to minor or major repair of existing mechanical equipment. A typical corrective maintenance task can be fixing a failure of a component which can considerably be a more significant task, compared with a typical predictive maintenance task, whereas it might, in some cases, only need readjustment of settings, e.g., setting CNC machines to better cope with a potential failure or a certain production process. In the case of dependent maintenance, conducting a maintaining task can be increased by the required task accommodating the dependent machine component, e.g., stopping the machine, and restarting it in line with the maintained machine equipment.

Algorithm 4

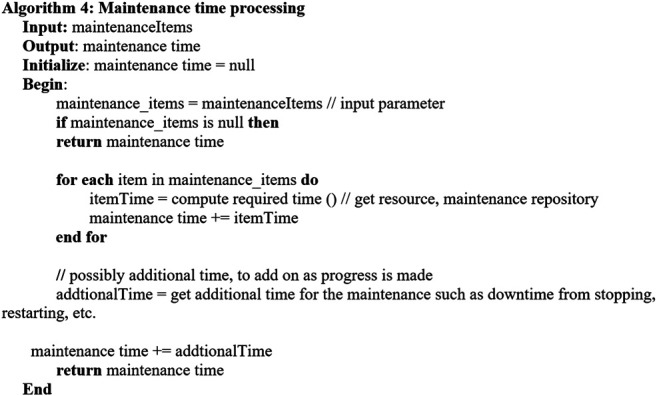

When the maintenance-required items with the corresponding tasks, i.e., repair/replacement, are determined, the maintenance time, i.e., Algorithm 4, is invoked.

Algorithm 4 describes maintenance time processing considering multiple machines with multiple components which require related maintenance.

The process takes outstanding maintenance items as the inputs. The maintenance time is determined by considering the maintenance activity time for each item from the resource repository which stores maintenance tasks, i.e., repair or replacement with associated maintenance time, e.g., 1 day. Moreover, additional time such as startup and shutdown is considered for the overall downtime and hence is added into the maintenance time.

Moreover, the maintenance time may also cover the time from preparation to conducting a maintenance task, the interval between stopping and restarting the machine, the time for a maintenance operator or engineer to conduct the maintenance task, and the actual time for the maintenance work of each machine component repair, replacement, readjustment, displacement, etc. The duration of maintenance can also depend on the condition status of maintenance tasks, i.e., failure and worn. The sum of these variables can be considered the overall downtime of a production line, affecting the whole manufacturing chain.

After the maintenance items with the associated maintenance task and maintenance time are processed, the *resource* required for the maintenance activity is determined using the resource repository. The resources such as the engineer, spare parts, and replacement items based on the nature of predicted failures are considered, and the required engineer/spare parts are to be assigned for the maintenance items.

Then, the availability of the maintenance items with associated resources, i.e., engineer, can be processed by invoking Algorithm 5 **.** *Availability* refers to the status, i.e., availability of the resources for a maintenance operation, of the machine equipment with associated components, i.e., spare parts or replacement item; processes, i.e., checking against the production plan; and a maintenance engineer/operator. This means that getting part of the availability information is coordinated with other activities, processes, etc., for an optimal maintenance task with minimal impact.

Algorithm 5

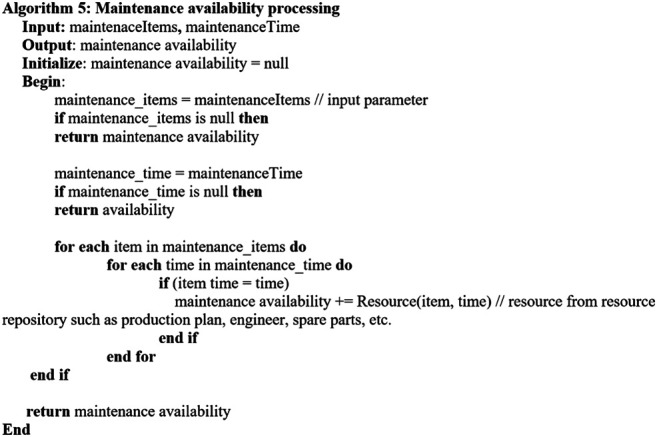

Algorithm 5 illustrates maintenance availability processing considering multiple machines with multiple components which require related maintenance.

The process takes the outstanding maintenance items and their availability as the inputs. They are then processed through the maintenance available slots from the resource repository. The maintenance available slots are determined by the maintenance RUL time window of the input maintenance items and the corresponding input availability.

Then, the maintenance related cost can be proceeded. Algorithm 6 depicts maintenance cost processing considering multiple machines with multiple components which require related maintenance. As inputs, the outstanding maintenance items with their corresponding maintenance time and cost are considered. The cost intends to support for any flexible or dynamic costs that incur for maintenance by accepting the fixed cost for any item as an input parameter.

Algorithm 6

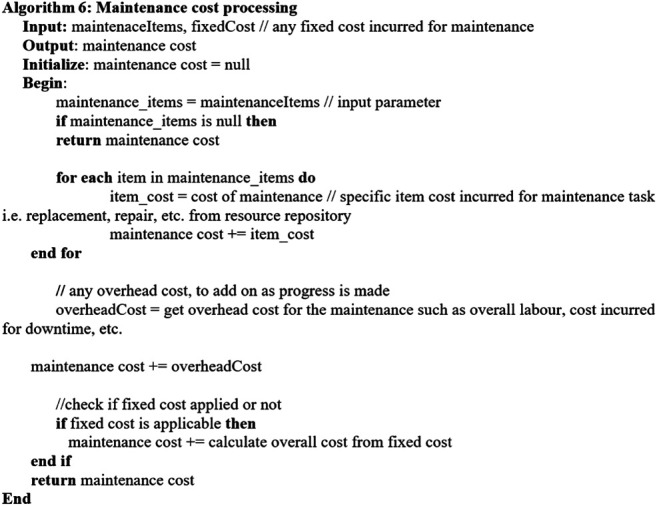

The maintenance cost is determined by a combination of maintenance item cost, i.e., repair or replacement, and maintenance time for performing the required activity or task. This maintenance-related information is accommodated by the resource repository. Moreover, overhead cost such as the cost of an engineer and setup and any dynamic cost as inputs are included in cost processing.

4. Then, the predictive maintenance schedule in Algorithm 2 is proceeded. The objective goal of the optimal maintenance schedule is to minimize the overall cost related to conducting the required maintenance and reducing downtime. The goal of the maintenance task, i.e., short, medium, or long term, may become critical, depending on the degree of the task, time, and cost. To achieve an optimal solution, the maintenance group concept is applied (Dekker et al., 1997). In this context, for a factory production line, the setup cost which is the cost of sending a maintenance team to the site, stopping the production, and resetting the production environment and fixed/maintenance cost for a maintenance visit to conduct the maintenance activities for several components in one joint maintenance interval, rather than for a single component, are considered. Then, the *PMS4MMC* process is run to get an optimal maintenance schedule which can subsequently be available for the decision-maker in their maintenance schedule plan.
5. To support the dynamic nature of the manufacturing network, i.e., different business requirements or changes, the PMS4MMC process supports handling new data, i.e., machine and maintenance, as illustrated in No. 5 of Figure 6. This means invoking the predictive maintenance schedule of Algorithm 2 and related processes No. 1–No. 4 of Figure 6 with the corresponding algorithms (Algorithms 3–6)*.* This enables using updated/new maintenance data, RUL values, etc., and adjusting appropriate optimization parameters to get the desired plan. Also, an optimized RUL model can also be re-tuned/deployed upon acquisition of new data.

### Predictive Maintenance With PMMI 4.0 and PMS4MMC

The contribution of this work is to provide an Industry 4.0 predictive maintenance model which is flexible enough to support the need of predictive maintenance services for a complex Industry 4.0 collaborative manufacturing environment. PMMI 4.0 thus is constructed for offering a coherent view of different key modules with related processes for implementing an effective Industry 4.0 predictive maintenance solution. To support complex Industry 4.0 systems, maintenance analysis can be done using PMS4MMC for maintenance scheduling based on the predictive RUL and maintenance-related data as demonstrated in *Predictive Maintenance Process and Predictive Maintenance Model for Industry 4.0* for assisting maintenance decision-making.

First, raw data generated from machine equipment tools, processes, and systems of the manufacturing factory must be collected and processed for analytics. The capability of big data and advanced analytics is enabled to facilitate the factory staff, e.g., operator, for making an effective maintenance-related decision. In the context of PMMI 4.0, maintenance data are stored in databases such as HDFS using the data model as illustrated in Figure 2 for better supporting maintenance. The maintenance repository stores related maintenance data including the existing maintenance schedule. Maintenance scheduling is facilitated by the data model for maintenance-, machine-, or resource-related data. These data are made available for decision-supported maintenance in assisting maintenance decisions.

In the case of assisting decision-supported maintenance in PMMI 4.0, the maintenance analysis is performed for creating a maintenance schedule plan as described in 4.2 and Figure 4. The outcome of the analysis determines the maintenance schedule plan for all the tasks and related activities that take into consideration the different weights such as cost. In this case, it can be computed as follows: the maintenance task is considered the estimated automation task or operator task for the assets, i.e., machine equipment tool which requires the corresponding maintenance activity. This can also depend on the maintenance time, e.g., displacement and repair, the relative position of the maintenance item, or the automation of the repair machine equipment of the completed maintenance activity. Moreover, the availability of the asset items for maintenance is considered. In this context, potential maintenance-required items are checked, e.g., work-in-progress production, and the corresponding qualified timeslot is considered. The cost refers to the cost associated with maintenance, i.e., repair, downtimes, and replacement, of the asset items. The costs also depend on the nature of the maintenance task as well as the technician or operator. Overall, the maintenance schedule activity is initiated by performing the maintenance analysis considering the described maintenance constraints such as cost and resources.

Also, different notifications regarding various critical maintenance asset conditions and maintenance analysis based on time, cost, and availability are also considered at this level. Using the alert notification of the machine condition of each asset including its future trend and RUL, the maintenance analysis is carried out for an optimal maintenance schedule plan with appropriate task activity.

## FIRST Flexible Manufacturing Case

In this section, we demonstrate and validate the proposed PMMI 4.0 and PMS4MMC using the FIRST project’s flexible manufacturing case. The manufacturing case and its implementation environment are explained in *Application Case* and *Implementation Environment*. Maintenance scenarios based on maintenance data from the FIRST industrial application case are used for the validation of PMS4MMC, and subsequent results are presented in *Maintenance Scenarios*.

### Application Case

**FIGURE 7:**
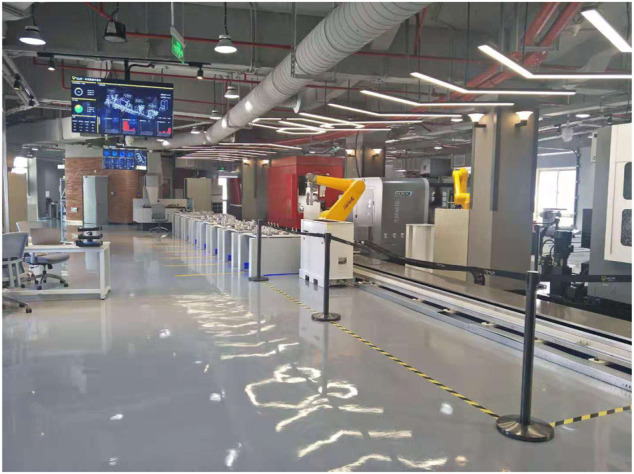
FIRST flexible manufacturing case (Sang et al., 2020).

A flexible manufacturing factory is facilitated by several different systems such as machine equipment tools, e.g., robots, processing systems, and supply chain management systems, and other auxiliary systems. In the industrial case used in this work, the factory processing system is facilitated by four sets of machines, three robots, several AGV trolleys, and carrier plates with a warehouse, as illustrated in Figure 7. Several machine tools including a coordinate measuring machine, cleaning machine, and drying machine are used for different operations, e.g., measuring, cleaning, and drying the workpiece. The operation of these different machine equipment tools produces various data which can be used for different analytical purposes. Furthermore, the factory operates with different processes and data from collaborative partners, i.e., machine manufacturers, suppliers, and insurers, in the manufacturing chain. This also means that different collaborative business processes exist for different business needs, and hence, data are being processed across different domains.

A universal tray is used for the workpiece with high re-positioning accuracy. This allows the ease of processing of the different workpieces to be quickly positioned and clamped in various involved equipment. Each workpiece is being identified with an RFID chip on the tray. When the workpieces are loaded on a carrier board, they are then moved into the rough machining area by an AGV.

Each workpiece is processed as required and then is moved to roughing equipment for rough machining using the robot. After roughing, the robot moves the workpiece for cleaning and drying. After cleaning and drying, the workpiece is then moved by the robot to the area for fine machining. Fine machining operates the same way as rough machining. The roughing finishing workpiece is placed into the machine using the robot. After processing, it is again placed for cleaning and drying.

In the quality control process, the finished workpiece is placed into the three-coordinate measuring machine by the robot. Upon test completion, further processes are carried out for the workpiece as required. If the quality control satisfies the result, the workpiece is transferred to a warehouse or to be packed using an AGV. If the quality control is not satisfied with the result, the workpiece may need to be processed again.

### Implementation Environment

To demonstrate PMMI 4.0 and PMS4MMC for FIRST, data processing and prediction is to be applied as described in *Predictive Maintenance for Industry 4.0*. In this context, FIWARE’s components such as Cosmos Big Data Analytics, i.e., Cosmos Spark for streaming with corresponding data storage such as HDFS and CraftDB (i.e., time series facilitated by QuantumLeap), predictive maintenance services such as the predictive RUL model and PMS4MMC deployed and consumed by API, Orion Context Broker accommodating the interactions and communications *via* NGSI APIs (with PEP Proxy, Keyrock, etc., for the security aspect), and maintenance analysis facilitated by Grafana (e.g., alert monitoring), Hive (for an ad hoc query), and Angular frontend application for accessing the predictive maintenance services along with related maintenance data from databases such as HDFS on PMMI 4.0 (Catalogue 2021; Developers 2021; FIWARE 2021; Hadoop 2021) are utilized. *Python* Keras TensorFlow backend (Goodfellow et al., 2016) for the predictive model and *Python* Pulp Optimization for PMS4MMC are utilized.

PMMI 4.0 is designed for supporting flexibility as described in *Architecture of Predictive Maintenance 293 for Industry 4.0*, and different or new business needs can be adapted into existing services or set up upon requirements. This can range from third-party software or commercial open-source tools (from FIWARE Marketplace) to GE components such as Knowage, which can easily be integrated into the platform, offering a potential solution for different business analytics (Catalogue 2021; Jason 2021). The next section provides the validation of PMMI 4.0 and PMS4MMC in a real-world industrial setting.

### Maintenance Scenarios

**FIGURE 8:**
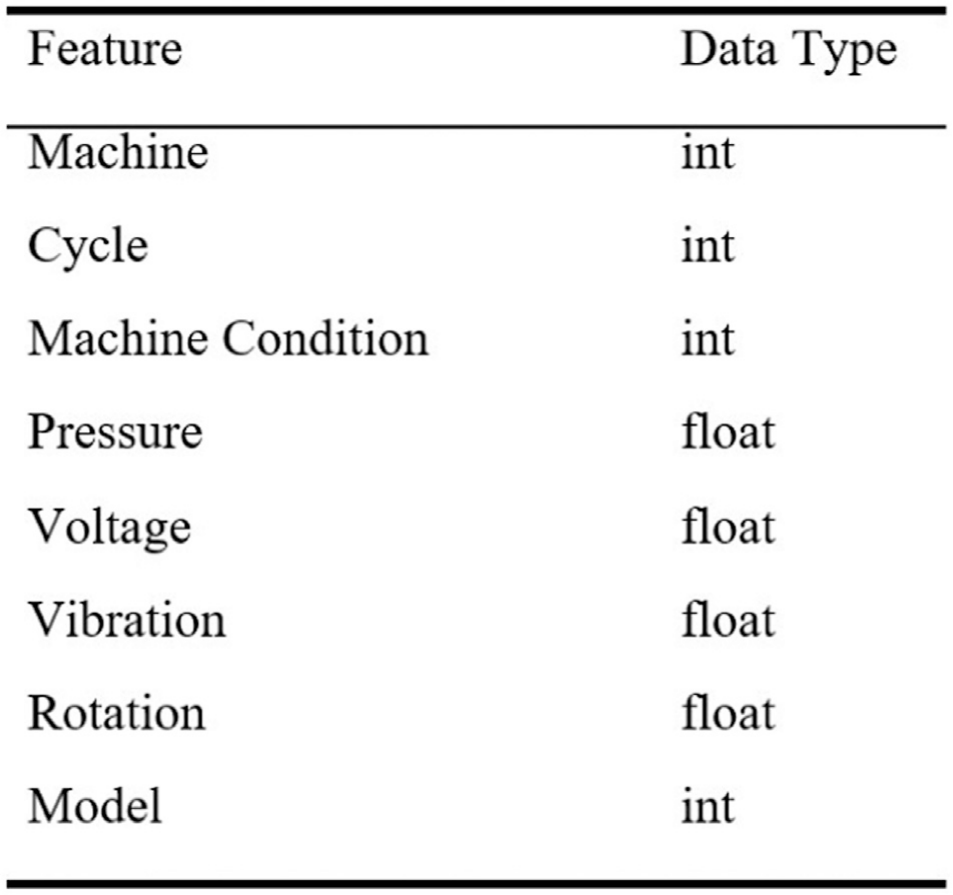
Sample data features from FIRST for training the predictive model.

To demonstrate the effectiveness of the proposed solution, the scenarios and data used must reflect or meet the nature and requirements of Industry 4.0, i.e., multiple machine components (i.e., *Predictive Maintenance for Industry 4.0*, *Approach for Industry 4.0 Maintenance Optimization*). In this sense, the maintenance and machine datasets (i.e., Figure 8 in *Predictive RUL Model* and Figure 10A in *Predictive Maintenance Schedule*) include different machine components with related data. To validate the dynamic nature of Industry 4.0 and business needs, two different scenarios with the consideration of multiple machine components and associated costs are initially established based on the maintenance data.

As such, this section presents the verification of PMS4MMC and predictive RUL model using the FIRST industrial dataset. In *Predictive RUL Model*, we present the results of the predictive RUL model (i.e., *Predictive Model for Maintenance*). In *Predictive Maintenance Schedule*, we describe the two scenarios used and present the corresponding results.

#### Predictive RUL Model

The prediction method, LSTM for RUL prediction described in *Predictive Model for Maintenance* and *Long Short-Term Memory Network (LSTM)*, is applied using the factory machine dataset from the application case. The machine dataset includes multiple machine components, operation data, and condition data collected during factory operation. The sample dataset feature used in this work is presented in Figure 8.

The predictive model is carried out following the requirement of the LSTM model in *Predictive Model for Maintenance*. Firstly, data processing is performed on the raw machine dataset to get appropriate input features using the variance threshold, RUL label. Data normalization is applied to the corresponding data, and the dataset is then processed to get both training and test sets (Goodfellow et al., 2016: Zheng et al., 2017). Upon completion of related data processing and LSTM requirements, the LSTM network model is built, defining and configuring the network parameters, i.e., the number of hidden layers, the number of neurons, and batch size. In this work, we used two LSTM layers with a fully connected neural network, which consists of hidden layers and neurons in every hidden layer (i.e., Figure 4 in *Predictive Model for Maintenance*). Regarding the training predictive model, the input refers to the training set and the target outputs are the actual RUL of the training set. To optimize the training network, the Adam optimizer is used with the learning rate set at 0.001 to achieve stable convergence. A high dropout rate of 20% was used after the LSTM or attention LSTM layer to combat overfitting. The Keras library with the TensorFlow backend is used for training the model (Goodfellow et al., 2016). Following the common evaluation approach for the RUL regression problem, i.e., related work in *Related Work* (Gers et al., 2003; Babu et al., 2016; Goodfellow et al., 2016; Zheng et al., 2017), the RMSE (i.e., root mean square error) is currently used as well as model comparisons with commonly used algorithms, i.e., *Model 1*—support vector regression (SVR), i.e., the machine learning method for time series prognostics (Chang and Lin 2011), and *Model 2—*convolutional neural network (CNN), i.e., the standard convolutional neural network (Babu et al., 2016).

**FIGURE 9:**
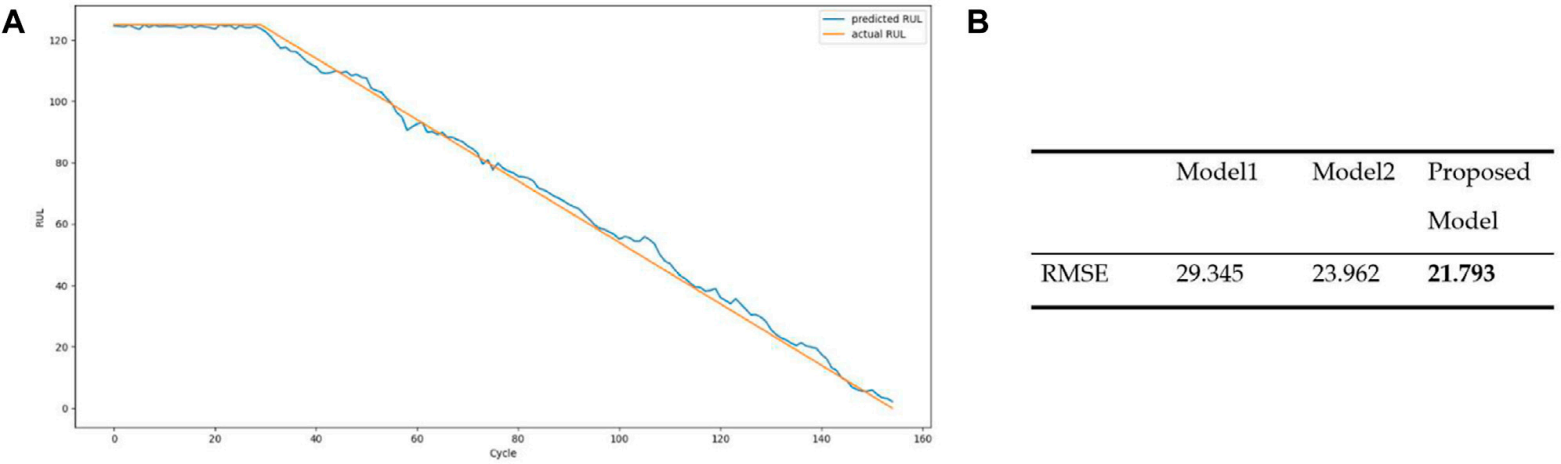
**(A)** The overall model predictions over the sample dataset depicting predicted and actual RULs. **(B)** Model performance (RMSE) comparison.

The results of training the RUL model utilizing LSTM (i.e., *Predictive Model for Maintenance*) are presented in Figure 9. Figure 9A depicts the sample dataset over the predicted (i.e., blue) and actual (i.e., green) data. Figure 9B shows the RMSE for the model against some commonly used regression models, Model 1 (SVR) and Model 2 (CNN). The current model performance, RMSE of our model, is over 21.793 which is not perfect but better than the others at 29.345 and 23.962, respectively. It could be improved with different networks/configurations/parameters as well as new sample data (Gers et al., 2003; Goodfellow et al., 2016; Zheng et al., 2017). Essentially, accurate RUL information of the machine in the later stage of its lifetime would provide actionable knowledge for effective maintenance management, reducing downtimes and costs. Using the predictive services, i.e., RUL model *via* FIWARE NGSI API, the RUL values of the machine component are then available for decision-makers in their maintenance schedule plan.

#### Predictive Maintenance Schedule

**FIGURE 10:**
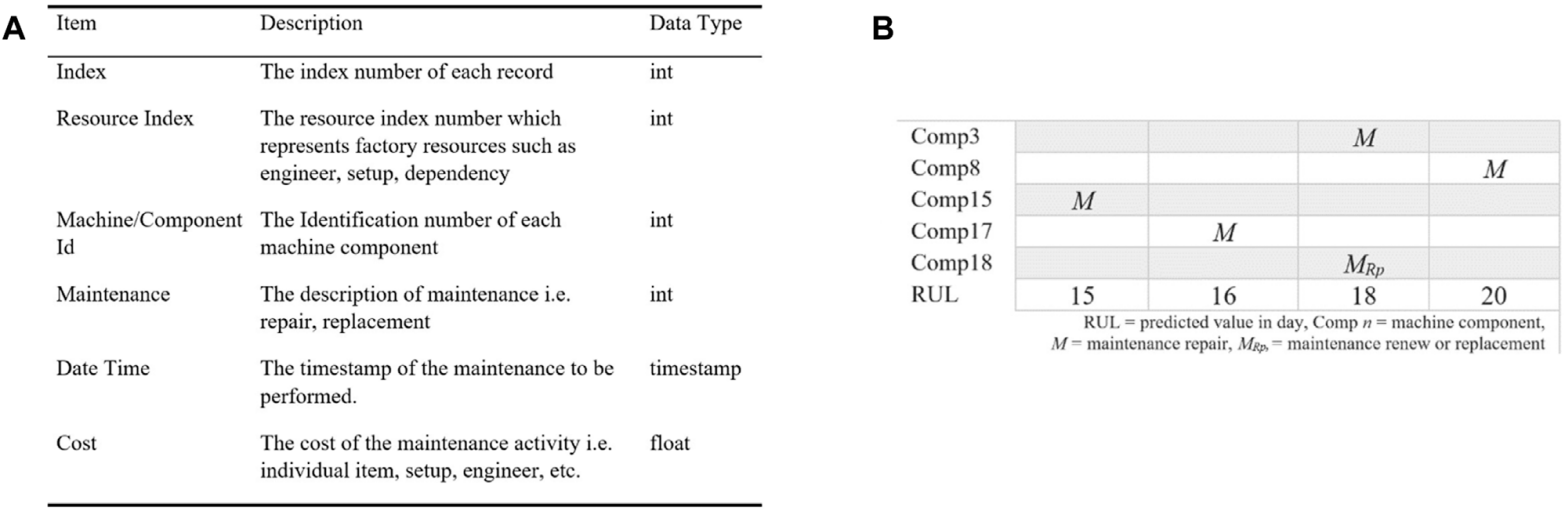
**(A)** Sample data for the predictive maintenance schedule. **(B)** Multiple machine components in the product line from FIRST depicting the respective RULs identified for maintenance analysis.

For *maintenance analysis*, we considered 21 components from one group of CNC machines in the product line of the FIRST manufacturing case. The maintenance of these multiple machine components includes the resource index (i.e., resource such as engineer), predicted RULs, maintenance tasks, timestamps, and related costs. The sample features used in this work are presented in Figure 10A. In the PMMI 4.0 context, these maintenance-related data (i.e., *Data Acquisition for Predictive Maintenance*, *Implementation Environment*) are being updated and stored using databases, i.e., HDFS, and are accessed *via* API, as illustrated in No. 2 of Figure 4.

Using the maintenance data, the RUL values, i.e., predicted value in days of the machine components, are identified over a time window of five-day period as illustrated in Figure 10B. In Figure 10B, RUL values for Comp 3, Comp 8, Comp 15, Comp 17, and Comp 18 are within 18, 15, 16, 18, and 20 remaining useful life (days), which are within a time window of five-day period. The decision-maker (e.g., maintenance engineer) can then use the maintenance items for initiating the analysis. This is assisted by the maintenance information which is available *via* the API (i.e., No. 2 of Figure 6). Regarding the maintenance schedule plan, the predicted five maintenance items (i.e., Comp 3, Comp 8, Comp 15, Comp 17, and Comp 18) with associated costs, resources (i.e., engineer), and the availability of the resources should be considered for allocating five different periods (i.e., five-day period) with two different options (i.e., during/after business hour), for the maintenance activities. In this case, four repairs and one replacement maintenance are considered as illustrated in Figure 10B. The maintenance activity, i.e., repair or replacement, can also be decided by a maintenance engineer based on the predicted RUL information and other related maintenance information, e.g., the availability of engineer.

In the case of constraints, all the machine components are scheduled within their RUL period to avoid substantial maintenance and related costs such as downtime and setup. The costs are extracted from the case data for this model. RUL values of the machine components are mostly utilized for scheduling as the cost of RUL is relatively less. Group maintenance, i.e., time window over 5 days with two maintenance time windows per day and optimizations such as maintenance engineer, and the availability of the resource and maintenance items based on the resource (i.e., *Approach for Industry 4.0 Maintenance Optimization*), i.e., factory location/dependency, are applied to reduce the high value of setup/location cost. This enables the model to minimize the number of setups with other associated costs including resource-based maintenance.

Two scenarios with a combination of inputs, i.e., different maintenance operation hours, and the different results are presented in *Predictive RUL Model and Predictive Maintenance Schedule*. In the first scenario, the maintenance is scheduled without the constraint of “the maintenance needs to be performed after business hour”; therefore, scheduling could cost a reasonably lower price. On the contrary, scenario 2 is planned during business hour and with notably higher associated maintenance costs. The maintenance costs such as engineer, setup, i.e., downtime of factory operation, and maintenance task, i.e., repair/replacement, are considered. The procedure of *predictive maintenance schedule* and the *algorithms* of maintenance optimization in *Proposed Predictive Maintenance Schedule for Industry 4.0 Multiple Machines and Components* are applied.

##### Scenario 1

**FIGURE 11:**
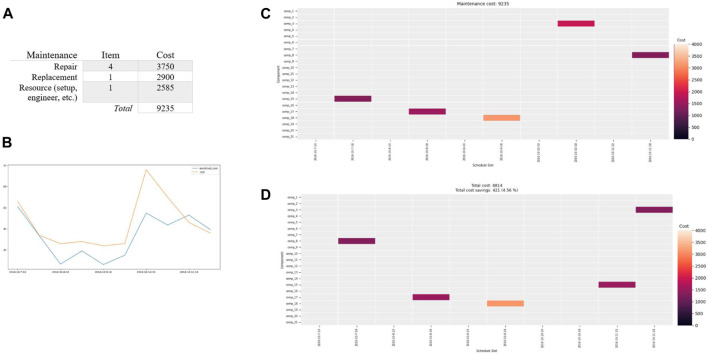
**(A)** The overall maintenance costs including resources of engineer and setup based on inputs, i.e., all maintenance 988 items for the five maintenance components over the five-day period. **(B)** Overall predicted cost comparison between the optimized cost (i.e., d) and the actual cost (i.e., c) over the same period. Maintenance schedule with group maintenance over the five-day period **(C)** without optimization and **(D)** with optimization over 4% cost-saving over the same parameters and period.

In the *maintenance analysis*, the input choices made by the decision-maker for *Scenario 1* include the resource costs including maintenance engineer, setup cost, i.e., shutdown/up factory machine, each item cost of the timeslot, and maintenance costs, i.e., repair/replacement, as illustrated in Figure 11A. The results are presented in Figure 11. Figure 11B depicts an overall predicted cost comparison between the optimized cost of Figure 11C (i.e., yellow) and the actual cost of Figure 11D (i.e., blue). The *x*-axis in Figures 11C,D shows different available schedule slots over the five-day period (with two different slots, i.e., during/after business hour), whereas the corresponding *y*-axis shows the multiple machine components for the maintenance scenario case.

##### Scenario 2

**FIGURE 12:**
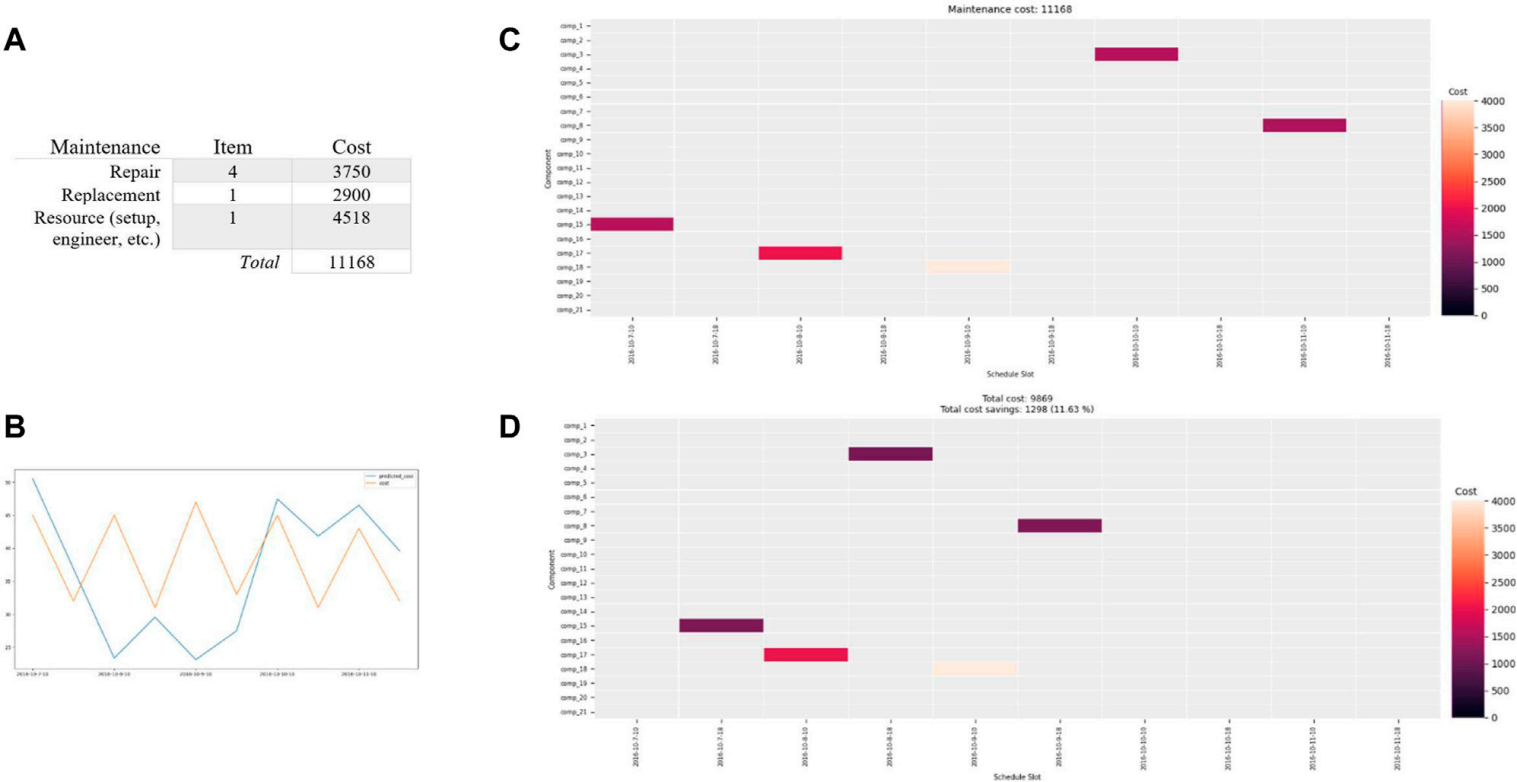
**(A)** The overall maintenance costs including resources of engineer and setup based on inputs, i.e., all maintenance items for the five maintenance components over the five-day period. **(B)** Overall predicted cost comparison between the optimized cost (i.e., d) and the actual cost (i.e., c) over the same period. Maintenance schedule with group maintenance over the five-day period **(C)** without optimization and **(D)** with optimization over 4% cost-saving over the same parameters and period.

Similarly, the same choices but after business hour and associated resource costs including maintenance engineer, setup cost, i.e., shutdown/up factory machine, each item cost of the timeslot, and maintenance costs, i.e., repair/replacement, are illustrated in Figures 12A,B for Scenario 2. The results are presented in Figure 12.

For *maintenance analysis*, both Scenarios 1 and 2 are available for the decision-maker in assisting planning as presented in Figures 11, 12. The results are based on dynamic options, i.e., based on RULs and inputs which illustrate options including five-day periods with different costs. The maintenance costs are also driven by constraints such as resource and availability. *Scenario 1* offers an option for the different time slots with a consideration of less resource, i.e., less cost (e.g., after business hour, resource, i.e., setup cost which covers the engineer and downtime of each group). On the contrary, *Scenario 2* offers different slots, including different resource constraints, i.e., costs. The output of *Scenario* 1 in Figure 11C offers no cost-saving, whereas Figure 11D presents over 4% cost-saving of the expected cost based on the five-day period window. Similarly, the output of *Scenario* 2 in Figure 12D offers over 11% substantial cost-saving of the same planning window, compared to Figure 12C and *Scenario 1*. The two comparisons, i.e., cost from both scenarios (i.e., Figures 11C,D, Fgures 12C,D), consistently illustrate that an overall predicted cost-saving indication can be made over the period if maintenance activity is performed as one optimal approach suggests. Ultimately, the maintenance engineer or operator can make appropriate maintenance decisions based on the business needs.

## Discussion

One of the challenges for Industry 4.0 manufacturing is to design and develop embedded services assisting in a flexible way the effective management of machine equipment tools by reducing downtimes and costs (Mobley 2002; Russell and Norvig 2010; Lee and Kao, 2014; Sang et al., 2020; Zonta et al., 2020; Sang et al., 2021). Our work focused on the design and development of a predictive maintenance model for Industry 4.0 (i.e., *Predictive Maintenance for Industry 4.0*) which utilizes the proposed predictive maintenance scheduling for multiple machine components (i.e., *Predictive Maintenance Schedule for Multiple Machines and Components*) by taking into account machine data such as operation, condition, and maintenance data. Through the application of big data analytics on new data streams in the connected machine equipment tools, the approach benefits from deep algorithms and optimizations to perform predictive maintenance.

In particular, we observed a gap in the application of both prediction models and maintenance optimization (Wildeman et al., 1997; Dekker et al., 1997; Van Horenbeek et al., 2010; Lee,et al., 2015a; Zheng et al., 2017; Al-Dulaimi et al., 2019; Sang et al., 2020), in the context of Industry 4.0, particularly for supporting scheduling of multiple machine components. Thus, PMS4MMC is proposed in *Predictive Maintenance Schedule for Multiple Machines and Components*. The FIRST industrial manufacturing case is used to demonstrate the validity of PMMI 4.0 and PMS4MMC in *FIRST Flexible Manufacturing Case* *.* In the scope of this work, we only used two different scenarios derived from the maintenance data. At this stage, it worked well based on the results (i.e., *Scenario 2*). However, constraints such as the complexity of Industry 4.0 manufacturing, domain knowledge, and limitation of the dataset, i.e., initial sample analysis, used in this work should be recognized.

Industry 4.0 focusing on manufacturing organizations such as the application case is complex, and this requires better understanding of complex processes, systems, etc. (Zezulka et al., 2016). This also means that there are several sophisticated structures, i.e., dependencies, configurations, and processes of different machine equipment tools (not just one organization but also multiple organizations). In this sense, the use of domain experts such as the factory manager, maintenance engineer, technician, and additional dataset including different sensor/operation/condition data as well as machine base from the machine’s manufacturers could certainly offer valuable inputs for improvements. In addition to the two scenarios used in this work, various scenarios such as high frequency of maintenance data, different resource constraints, more detailed maintenance task such as specific maintenance type of high cost, specific machine type with multiple components, etc., will improve the solution. This certainly requires accessing the knowledge of domain experts, maintenance data such as different levels of maintenance, task, type, operation, etc., as well as integrating data from different other information systems such as ERP and CMS. Additional optimization of the algorithms such as complexity analysis and evaluation, i.e., commercial software such as Gurobi, and reinforcement learning–based optimization will also be considered. This will provide further validation of the maintenance schedule process as well as performance tuning.

Industry 4.0 manufacturing also operates with several automated machines/tools, i.e., cyber–physical systems, manufacturing cell, robots, and smart devices (Bagheri et al., 2015; Lee et al., 2015b; Zezulka et al., 2016; Thoben et al., 2017). For instance, the manufacturing case used in this work also operates with three robots, with minimal intervention from factory staff such as operators. This offers further opportunities in acquiring the data for additional analysis of our work. This means that the orchestration of managing each predicted failure and maintenance scheduling will potentially and dynamically be managed by each robot itself with the help of advanced techniques such as deep reinforcement learning. We envisage this and introduce an initial maintenance process (i.e., automation) as part of maintenance monitoring in *Maintenance Monitoring*. This process incorporated with the proposed predictive RUL model and maintenance scheduling, i.e., PMS4MMC in *Predictive Maintenance Schedule for Multiple Machines and Components*, needs further work, and subsequent improvements can be made over our current work.

In the context of predictive models such as RUL estimation, traditional approaches such as model-based and experience-based cannot meet the demands of Industry 4.0 focusing on manufacturing. Thus, a data-driven approach using LSTM is adopted. The LSTM model is developed using the sample manufacturing machine dataset. At this stage, we only looked at the available sample dataset, though the current model is shown to be consistent, especially the machine is close to a failure. To get a better model, we plan acquiring additional dataset for evaluation and improvements. Using new sample data (i.e., different types, configurations, structures of machine components) from additional factory and machine operation/condition data will improve the model performance (Lei et al., 2018). In this sense, the utilization of new data collected from the machine equipment tools or new sample existing data including different types of machines/sensors from the application case will certainly offer significant improvements to our work. Model optimization may also be achieved by learning with different network layers/settings in the evaluation (Goodfellow et al., 2016). Furthermore, exploring sensor/data fusion and different networks/configurations/layers and integrating with transformer learning, another technique which is effective in sequence learning is also considered for our next work (Raffel et al., 2020). Thus, several tasks including acquiring new data samples, model tuning, validation, and re-deployment will be carried out.

In the context of predictive maintenance for Industry 4.0, existing solutions such as Lee et al., Chiu et al., Wang et al. (Lee et al., 2015b; Chiu et al., 2017; Wang et al., 2017; Schmidt and Wang 2018) do not consider the aspect of flexibility or modular platform, which is essential to operating complex and dynamic Industry 4.0 systems. Our solution, PMMI 4.0, however fits well with the FIRST industrial application case in achieving a modular platform with high interoperability and capabilities such as a big data analytics (Cosmos GE) component. In this sense, the embedded predictive maintenance services such as PMS4MMC into the existing systems can easily be adapted to different needs or can be integrated with different system processes. Security concerns such as privacy and encryption can also be managed with GE components such as Keyrock and Wilma (Catalogue 2021) or third-party tools. On the contrary, the implementation of FIWARE is generally based on an event-driven approach, and potential challenges such as increased complexity and security risks should be recognized and managed appropriately. The evaluation of commercial platforms such as Azure and Amazon with PMMI 4.0 for similar or different cases would also be considered for future work.

The focus of this work is the Industry 4.0 collaborative manufacturing context. Besides, PMMI 4.0 and PMS4MMC may well be applied to other industries, since big data analytics–enabled predictive capability/services become one of the key assets to organizations (Porter and Heppelmann 2014; Sang et al., 2016, 2017; Zezulka et al., 2016). For instance, a data center company may apply PMMI 4.0 by configuring the hard drive system with sensor devices *via* FIWARE’s IoT adapter connecting streaming processing with the Cosmos Big Data Analytics GE component for maintenance purposes (Catalogue 2021; Jason 2021). A predictive RUL model for the hard drive can also be developed and configured with PMS4MMC, and subsequently, an optimal predictive maintenance schedule can be made appropriately. Furthermore, similar adoptions may well be applied to other industries such as virtual factories and smart cities, e.g., electricity station or traffic light for sensor monitoring and maintenance purposes.

## Conclusion and Future Work

In this work, we designed PMMI 4.0 (i.e., *Predictive Maintenance for Industry 4.0*), a predictive maintenance model for Industry 4.0 which utilizes the proposed PMS4MMC (i.e., *Predictive Maintenance Schedule for Multiple Machines and Components*) for supporting a predictive maintenance scheduling–driven LSTM RUL model. FIRST’s industrial manufacturing case is used to demonstrate the effectiveness of PMMI 4.0 and PMS4MMC implementing FIWARE Cosmos Big Data Analytics to support a flexible Industry 4.0 platform in *FIRST Flexible Manufacturing Case*. Factory operation and maintenance datasets are used for illustrating PMS4MMC which achieved over 11% optimization (i.e., *Predictive Maintenance Schedule*). This demonstrates the real-world application of the model in an Industry 4.0 context.

As highlighted in the discussion (i.e., *Discussion*), further improvements and optimizations of PMMI 4.0 and PMS4MMC remain as our future work, which includes further enhancement, i.e., maintenance and model with additional/new datasets, different methods, i.e., different network layers/settings for the predictive model, different scenarios (i.e., frequency/level/type/constraint) of maintenance scheduling, and other use cases across industries, in particular in relation to different manufacturing modes (i.e., discrete vs. continuous manufacturing). The considered industrial cases include virtual factories or complex collaborative network organizations, whereas predictive maintenance services are monetized and offered. For future research, as the industries are increasingly embracing the concept of Industry 4.0, several directions including dynamic maintenance, i.e., self-maintenance/automation of prediction and scheduling optimization for complex smart machine tools, as well as related optimizations of algorithms or processes applying big data analytics in providing an effective predictive maintenance, could be considered.

## Data Availability

The original contributions presented in the study are included in the article/supplementary material, and further inquiries can be directed to the corresponding author.
